# Integration of single-cell multi-omic data with graph-based topic modelling

**DOI:** 10.1093/bib/bbag608

**Published:** 2026-11-15

**Authors:** Gabriele Malagoli, Filippo Valle, Andreina Tirabassi, Annalisa Marsico, Loredana Martignetti, Michele Caselle, Maria Colomé-Tatché

**Affiliations:** Institute of Computational Biology, Computational Health Center, Helmholtz Munich, Ingolstädter Landstraße 1, D-85764 Neuherberg, Germany; Biomedical Center, Division of Physiological Chemistry, Faculty of Medicine, Ludwig-Maximilians-Universität Munich, Großhaderner Str. 9, 82152 Planegg-Martinsried, Germany; Physics Department, University of Turin and INFN, Via Pietro Giuria 1, 10125 Torino, Italy; Institute of Computational Biology, Computational Health Center, Helmholtz Munich, Ingolstädter Landstraße 1, D-85764 Neuherberg, Germany; TUM School of Computation, Information and Technology (CIT), Boltzmannstraße 3, 85748 Garching bei München, Germany; Institute of Computational Biology, Computational Health Center, Helmholtz Munich, Ingolstädter Landstraße 1, D-85764 Neuherberg, Germany; Institut Curie, Inserm U1331, Mines ParisTech, PSL Research University, 26 rue d'Ulm, 75248 Paris cedex 05, France; Physics Department, University of Turin and INFN, Via Pietro Giuria 1, 10125 Torino, Italy; Institute of Computational Biology, Computational Health Center, Helmholtz Munich, Ingolstädter Landstraße 1, D-85764 Neuherberg, Germany; Biomedical Center, Division of Physiological Chemistry, Faculty of Medicine, Ludwig-Maximilians-Universität Munich, Großhaderner Str. 9, 82152 Planegg-Martinsried, Germany; Hospital del Mar Research Institute (HMRIB), C/Doctor Aiguader 88, E-08003, Barcelona, Spain

**Keywords:** single-cell multi-omics, topic modelling, stochastic block model, multipartite networks, gene regulatory programs, data integration

## Abstract

Recent advances in single-cell biology enable the profiling of multiple molecular layers, such as the transcriptome, epigenome, and surface proteins, within a single cell. Tackling the complexity of these data from multiple perspectives allows researchers to gain the most comprehensive insights into cellular biological properties. Here, we propose a graph-based topic modelling method called bionSBM. Our method leverages well-known community-detection methods for multipartite graphs and the interpretability of topic modelling to cluster and explain high-dimensional, sparse, and noisy single-cell matrices. We applied our algorithm to paired single-cell multi-omics data, such as 10X Multiome, SHARE-seq, and CITE-seq. We showed that it achieves superior performance compared with state-of-the-art methods for ground-truth label retrieval, with high specificity and distinct biological interpretability.

## Background

The emergent phenotypes of biological systems result from complex molecular interactions. These interactions play a role at multiple levels of gene expression regulation: genome, epigenome, transcriptome, and proteome. Characterizing such regulatory behaviour is crucial to studying these processes. It is an essential step towards the development of drugs and targeted, personalized medicine strategies [1], as well as for other translational applications such as drug discovery and validation [2] or tumour microenvironment profiling [3]. The latest advances in single-cell sequencing technologies, such as 10X Multiome and SHARE-seq [4], enable profiling of gene expression and chromatin accessibility at the single-cell level. Moreover, technologies such as CITE-seq [5] allow profiling not only of gene expression but also of surface molecules using antibody-derived tags (ADTs). All these techniques are opening new avenues for understanding complex gene regulatory mechanisms [6].

The development of experimental protocols poses a new challenge for the scientific community: algorithms and downstream pipelines must be adapted to integrate paired and multi-omic data. In particular, when analysing these data, one is typically interested in their modular structure. The main goal is to identify groups of molecular features that act in a coordinated way, as well as groups of cells with similar molecular patterns. Given the multimodal nature of the data, the focus can be on gene groups, ATAC-seq peaks, ADTs, or any features captured by the measurements. Moreover, it is of interest to link these modular structures across omics layers and use them to infer cell-level clusters, a highly non-trivial task. Several algorithms have been proposed in recent years to address this task. A popular approach is deep learning. Deep learning methods, such as autoencoders [7], perform effective dimensionality reduction but are sensitive to differences in input scale, a key characteristic of multimodal data [8]. More recent approaches have extended representation learning to increasingly heterogeneous settings, including contrastive learning for single-cell mosaic integration in ACE [9] and graph neural networks combined with contrastive learning for spatial multi-omics in SpaMosaic [10]. Network-based integration methods provide another strategy and differ substantially in how omics measurements are represented as graphs. For example, NIC learns modality-specific cell–cell similarity networks from transcriptomic and epigenomic profiles and integrates them through joint matrix factorization [11], whereas other multi-network approaches construct gene–gene networks from protein interactions and methylation data [12], or from differential co-expression and co-methylation patterns [13].

Here, we focus instead on a class of algorithms based on topic modelling. Topic modelling algorithms were initially developed in natural language processing (NLP) to automatically identify ‘topics’ in a corpus of texts, using only word frequencies as input. They can group sets of words that ‘speak about the same theme’ and, in this way, identify the texts’ topics. There is an obvious analogy between this task and the one we are interested in. If we map texts to cells, and the frequency of words to gene expression levels, chromatin accessibility at ATAC-seq peaks, or surface protein levels, the same approach described above can be used to identify and characterize cell populations based on their patterns of molecular measurements. In this analogy, topics are groups of genes/open genomic loci/surface proteins that act cooperatively, and these regulomes and pathways can be used to characterize different cell populations. The rationale for using topic modelling methods for single-cell data is that the cell transcriptome exhibits statistical properties similar to those of words in text [14]. One of the most interesting features of topic modelling algorithms is that they output probability distributions over cells for each topic, which better describe the complexity of biological systems than a naive, deterministic assignment in which cells are simply assigned to a group (or cluster). The topics, on the other hand, are not only lists of loci; they are described as probability distributions over all genomic features.

The most popular topic models are based on Latent Dirichlet Allocation (LDA) [15], Non-Negative Matrix Factorization (NMF) [16], or Stochastic Block Models (SBM) [17]. LDA approaches the problem of topic modelling by assuming a Dirichlet prior for the distribution of words within topics and of topics within documents (in our setting, corresponding to the distributions of genes, peaks, and ADTs within topics, and of topics within cells). The choice of this prior is mostly for analytical and computational reasons, lacking a real biological motivation [18]. Since the Dirichlet distribution is unimodal, regardless of the choice of hyperparameters, it assumes a single shape for the distribution of words across topics and topics across documents, which is a strong homogeneity assumption that is difficult to reconcile with the heterogeneity of biological systems [19]. On the other hand, NMF has two main limitations: its linearity limits the ability to capture complex interactions, which are typical in biological systems, and it tends to find steep boundaries between clusters, which can be problematic when dealing with highly heterogeneous data, as in single-cell sequencing [18].

We will therefore concentrate on the following in the third category of topic models, based on SBM [17, 19, 20]. We present a graph-based method, bionSBM, for integrating and interpreting paired single-cell multi-omic data [Fig. 1]. In contrast to approaches that first derive cell–cell or gene–gene similarity networks, bionSBM directly represents each modality as a cell–feature bipartite layer, in which each non-zero molecular measurement defines a weighted edge between a cell and the corresponding feature. These layers share the cell nodes and are jointly modelled as a single multipartite network. bionSBM can cluster cells based on any modality a user deems relevant (peaks, ADTs, messenger RNAs (mRNAs), the complete transcriptome, etc.), treating them symmetrically *a priori*, meaning no modality-specific weights or scaling coefficients are introduced. It does not impose a unimodal prior; instead, it assumes a uniform prior to fit the topics-cells and features-topics distributions. In [21], it was shown that a uniform prior can yield better results even with non-uniform synthetic latent variables. Moreover, in [19], it was shown that the prior assumptions of different algorithms can affect the inferred results; therefore, a uniform assumption is the one with as little information as possible. This algorithm is part of a long-standing effort to apply SBM methods to biomedical data, which started with the construction of a hierarchical version of SBM-based topic modelling (hSBM) [18] for gene expression data, then expanded to bulk tissue multi-omics data (nSBM) [22], to single cell gene expression data [23] and finally, in the present paper, to single cell multi-omics data. In the following text, we first describe the main features of bionSBM, then compare it with the topic modelling integration algorithms ShareTopic [24] and Mowgli [25], as well as COBOLT [26], MultiVI [27], and totalVI [28].

**Figure 1:**
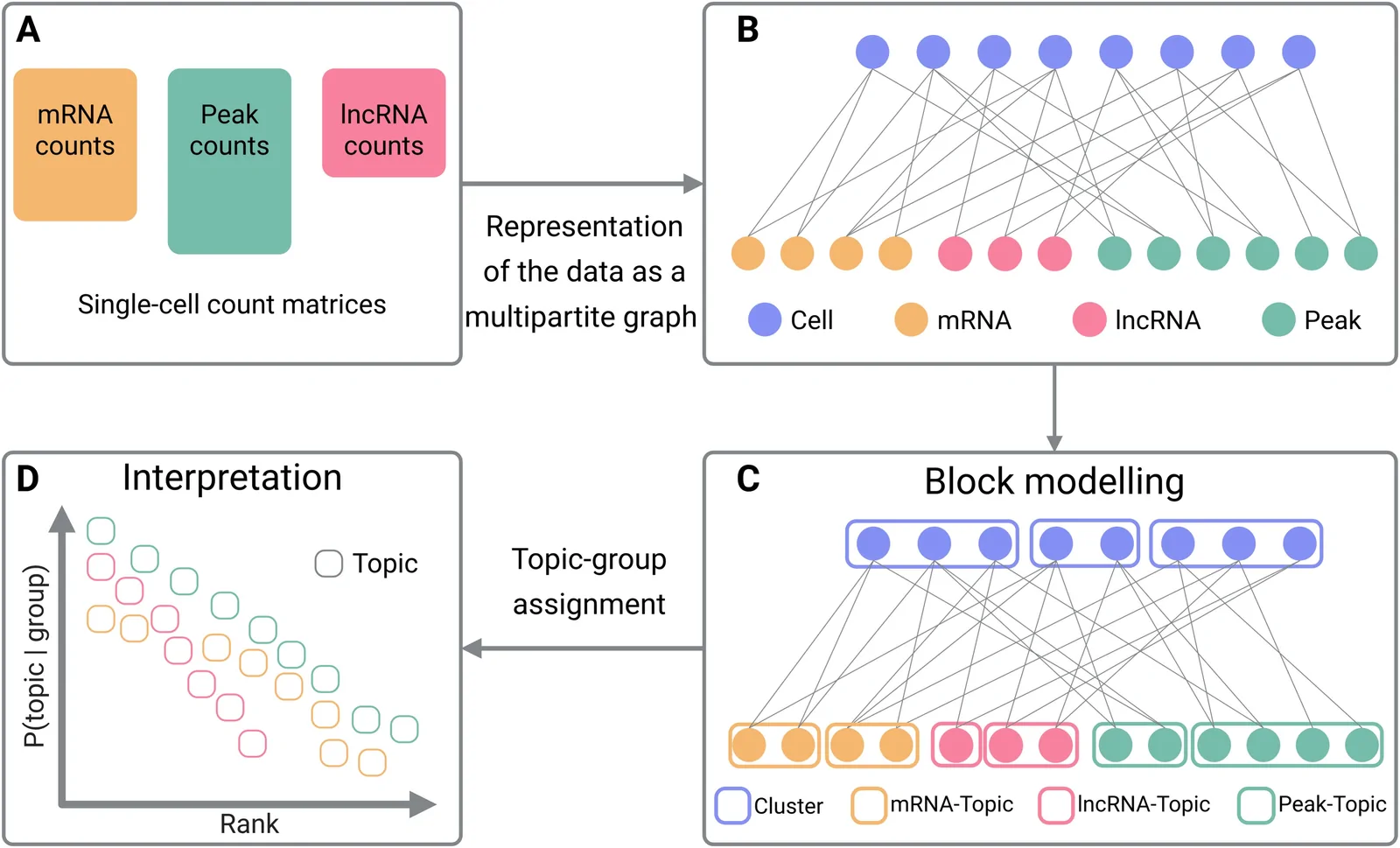
Schematic pipeline of the bionSBM algorithm. **A** Data are input as count matrices. **B** Input matrices are used to build a multipartite network: features are connected to the cell branch if the feature is expressed/open in the cell with weighted edges, but features are not connected. **C** bionSBM finds the best block model to group the cells into clusters and the features into topics. **D** The explanation for the cell grouping can then be easily obtained from the topic distribution probabilities within each cluster. Figure created with BioRender.

## Results

### The bionSBM model

bionSBM leverages a class of algorithms known as SBMs [17]. In particular, bionSBM is based on a remarkably efficient hierarchical version of SBM, developed in [19, 20], known as hierarchical stochastic block modelling (hSBM), which was later extended to n-partite SBM (nSBM) for multipartite graphs.

The idea behind hSBM is to maximize the posterior probability of the model given the data, namely *P(model ∣ data)*. In a Bayesian framework, this is done by assuming a model *P(model)* and maximizing the likelihood *P(data ∣ model)*. hSBM assumes a uniform prior on *P(model)* and performs statistical model selection based on the principle of minimum description length (DL) (Methods). In general terms, this class of algorithms seeks the most compact possible representation of the data.

In the context of single-cell omics data, the observations correspond to molecular measurements, such as single-cell RNA sequencing (scRNA-seq) or single-cell ATAC-sequencing (scATAC-seq) matrices [Fig. 1A], and the model is a hierarchy of partitions of both molecular features and cells. In previous work, hSBM was extended to nSBM [22] to accommodate a data structure with multiple sources. Now, the bionSBM algorithm is efficiently implemented to handle a multipartite network built from single-cell data, where nodes represent different entities, such as genes, peaks, or proteins, and cells [Fig. 1B]. Genes and peaks are connected to cells based on their molecular measurement levels, using weighted edges. bionSBM allows multiple feature types to be associated with the same data point (i.e. cell) [Fig. 1B]. This enables the concurrent modelling of multiple modalities in a single inference run [Fig. 1B, C]. bionSBM is integrated into the single-cell Python universe [29] by handling *AnnData* [30] for single-modality data and *Muon* [31] objects for multi-modal data.

In addition, bionSBM distinguishes itself from nSBM in terms of time and scalability by efficiently creating large sparse graphs, which are characteristic of single-cell experiments [Supplementary Fig. S1 A, B]. A key advantage of bionSBM over other algorithms is that, because it operates on graphs, preprocessing does not require scaling or harmonizing values across omics data. Moreover, features with heterogeneous statistical characteristics (counts, continuous values, or binary measurements) originating from different omics layers and preprocessing pipelines can be incorporated directly into the multipartite graph, as the inference procedure does not require cross-modality scaling or harmonization. The output of bionSBM is a hierarchical clustering of cells, while providing modality-specific topic assignments [Fig. 1D]. In other words, the terms ‘clusters” and ‘topics” assume a different meaning than in LDA-based approaches: here, a cluster is a set of cells, like in a community detection method, but a topic is a set of features. This allows the creation of groups of cells (clusters) [Fig. 1C], groups of peaks (peaks-topics) [Fig. 1C], groups of genes (gene-topics) [Fig. 1C], or any other kind of feature, for the same multipartite graph. This richer representation allows the disentangling of distinct feature sets across modalities. It is important to note that bionSBM requires that nodes of a given type be non-mixed, so that blocks contain only homogeneous nodes; hence, each group will contain either cells, genes, peaks, etc.

### Model benchmarking

We compared the performance and results of bionSBM with those of ShareTopic [24], Mowgli [25], COBOLT [26], MultiVI [27], and totalVI [28] on six different data sets. We chose them to represent a variety of situations, including mouse and human genomes, from fully developed to differentiating cells. The data sets show a heterogeneous complexity, ranging from a few thousand to 40 000 cells and from about 10 to 35 cell types. The number of genes per experiment is one of the most homogeneous characteristics across data sets, because the species fix it [Supplementary Fig. S2]. Four of the data sets considered (PBMC from the 10x database [32], BMMCMultiOme [33], HSPC [34], MouseSkin [4]) consist of gene expression and chromatin openness data (scRNA-seq and scATAC-seq). Among these, the MouseSkin data set has been profiled using the SHARE-seq technology [4], whereas the other three are based on the 10X Multiome platform. Two additional data sets, Spleen [28] and BMMCCite [35], are CITE-seq experiments that combine gene expression and surface protein data. This ensemble of diverse data was chosen to test the efficacy of the methods across different contexts, ensuring that performance is not driven by technological, species-, or omics-level biases (see Supplementary Table S1, Methods).

For benchmarking, ShareTopic can handle only scATAC-seq and scRNA-seq pairs; therefore, the two Cite-seq data sets are excluded from this method. For the other four data sets, we could compare all the algorithms. For each data set, we explored different options for inputting transcriptional features. In a previous study [23], we showed that cell classification improves when analysing the expression of protein-coding genes and long non-coding RNAs (lncRNAs) as separate omics layers using the aforementioned nSBM approach. Hence, we created three transcriptomic count matrices: the total Gene Expression (GEX), which contains all the possible transcripts measured by an scRNA-seq experiment; the ‘mRNA’ matrix, containing only protein-coding genes; and the one named ‘lncRNAs’, which consists only of lncRNAs.

We tested the quality of the results on the cell layer separately (to evaluate the algorithm’s ability to recover cell types) and on the feature layers (to assess topic quality across different feature spaces). For each test setting, we ran the algorithms multiple times to explore the stochasticity of the results and the impact of various combinations of transcriptional features. All the algorithms were executed 25 times per data set and feature-space combination, and we measured runtime and memory usage to provide an accurate report of the required resources [Supplementary Fig. S1 C, D].

All the methods embed the input spaces into a low-dimensional representation. Mowgli and ShareTopic create a single low-dimensional topic space, where each topic is a mixture of features across all omics, making interpretation more laborious because the algorithm mixes features from different omics; the same applies to COBOLT factors. Instead, bionSBM treats each -omic separately in its own output. Therefore, when using the term ‘topic’ for bionSBM, we will specify which -omic we refer to (mRNA-topic, peaks-topic, etc.), while for ShareTopic, Mowgli, and COBOLT, the topics will be a mixture of these. MultiVI and totalVI do not create topics or factors.

### bionSBM better recapitulates cell type in all the designed experiments and across technologies

We first evaluated the performance of the algorithms in recovering different cell types across the data sets. All the methods require the number of desired topics (or the latent dimension of the number of factors) as input. In contrast, bionSBM automatically infers the optimal granularity of each level of the hierarchy. The same applies to the number of clusters. In this work, for comparability, this hyperparameter was set to match the number of annotated cell types, thereby constraining the model to the expected biological heterogeneity. Notably, varying the number of topics/factors/latent dimensions of the comparator methods had little impact on performance [Supplementary Fig. S3].

To measure agreement between the clusters obtained with the algorithms and the ground-truth annotations across the six data sets, we show the NMI/NMI* (Methods). The larger the NMI/NMI* ratio, the better the agreement. Since this measure compares the clustering results to a random assignment of cells to clusters, it is affected by the characteristics of the specific data set, and the results should not be used to compare data sets (for instance, the number of cell types varies widely across data sets). Since bionSBM builds a hierarchy of partitions with different granularities, we suggest the user focus mainly on the one with the highest concordance with the known annotation, which is the one we report in Fig. 2. To make the effect of this *post hoc* selection explicit, Supplementary Fig. S4 reports NMI/NMI* across all inferred hierarchy levels for every dataset and experiment. The results show that high performance is often maintained across adjacent levels, highlighting that the choice of the level does not significantly affect the results. bionSBM shows performance similar to or better than the other methods [Fig. 2]. The PBMC data set is the one in which bionSBM stands out more significantly [Fig. 2A], whereas its performance on HSPC, BMMCMultiOme, and MouseSkin is less pronounced [Fig. 2B, C, D]. On the other hand, bionSBM achieves very high NMI/NMI* values on the CITE-seq dataset, even better than totalVI, which is tailored to these specific data sets [Fig. 2 E, F]. It is interesting to notice that the Cite-seq data also have the highest number of cell types, reflecting superior performance on more complex data.

**Figure 2:**
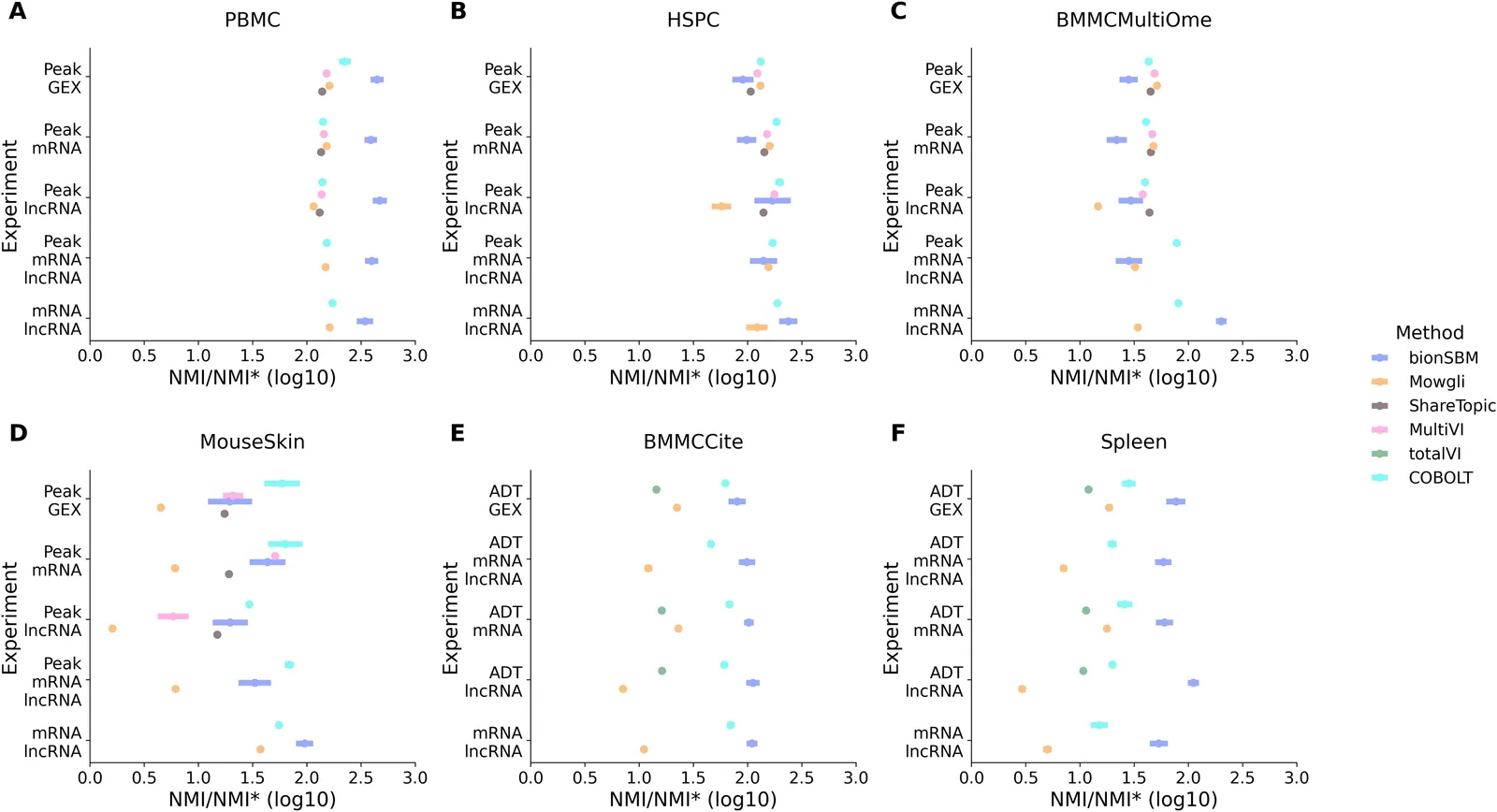
**A**–**F** NMI/NMI* score between each algorithm partition and ground truth annotation for the six data sets. Each dot represents the mean value, and the error bar represents three times the uncertainty on the mean. The higher the values, the closer the clustering is to the ground truth annotations.

### Graph-based topic modelling signatures are highly specific and clearly distinct

An important feature of algorithms in the topic modelling framework is their probabilistic output, encoded in the two probability distributions *P(topic ∣ cell)* and *P(feature ∣ topic)*, where feature is the biological feature (mRNA, lncRNA, peak, ADT) and topic is the appropriate topic family (mRNA-topic, etc). It is then possible to associate to each group of cells its most important topic simply using $t^{*}(group) = \arg \max _{t} P_{\mathrm{c}}(topic \mid group)$, where $P_{c}$ is a corrected version of the *P(topic ∣ cell)* (Methods). Moreover, one can assess the quality of this topic assignment using two standard metrics: specificity and distinctiveness. The former metric measures how group-specific the dominant topic is (i.e. the topic with the highest probability), while the latter captures how well topics are separated by quantifying the in-group and out-of-group topic probabilities (Methods). Both metrics take values in the range [0, 1], with 1 representing the maximum performance. We will assess them, excluding the AE-based methods, since they do not produce factors or topics. As mentioned above, bionSBM embeds each feature (mRNAs, lncRNAs, scATAC-seq peaks, etc.) in separate spaces, whereas Mowgli, ShareTopic, and COBOLT create a set of topics that mix features. Hence, the specificity and distinctiveness of bionSBM are computed for each -omic space, while for the other methods, the topic space is mixed.

bionSBM generally achieves high topic specificity [Fig. 3], higher than the other methods. The specificity is high across all data sets and modalities, indicating that the method is robustly able to explain each cell type in each modality. In agreement with the classification results [Fig. 2], bionSBM achieves a particularly high specificity in the BMMCCite and Spleen data sets [Fig. 3E, F], which are the most complex ones in terms of cell types [Supplementary Table S1]. The algorithms achieve similar values across all data sets for their distinctiveness metric [Fig. 4], with good performance in general, around 0.75.

**Figure 3:**
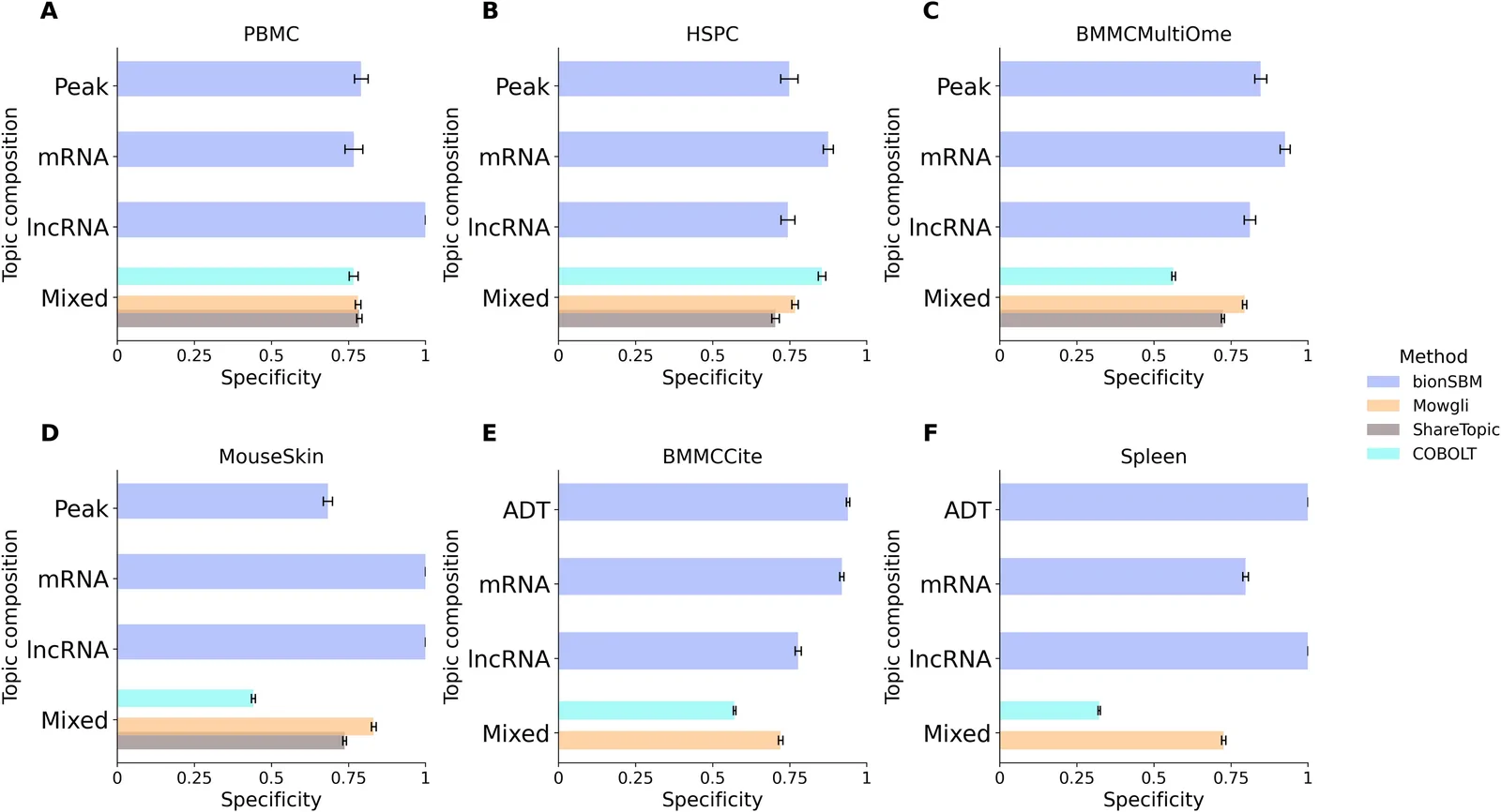
**A**–**F** specificity, defined as the fraction of cell groups assigned distinct dominant topics, for the six data sets. Each bar represents the mean value over multiple runs, and the error bar represents three times the uncertainty on the mean.

**Figure 4:**
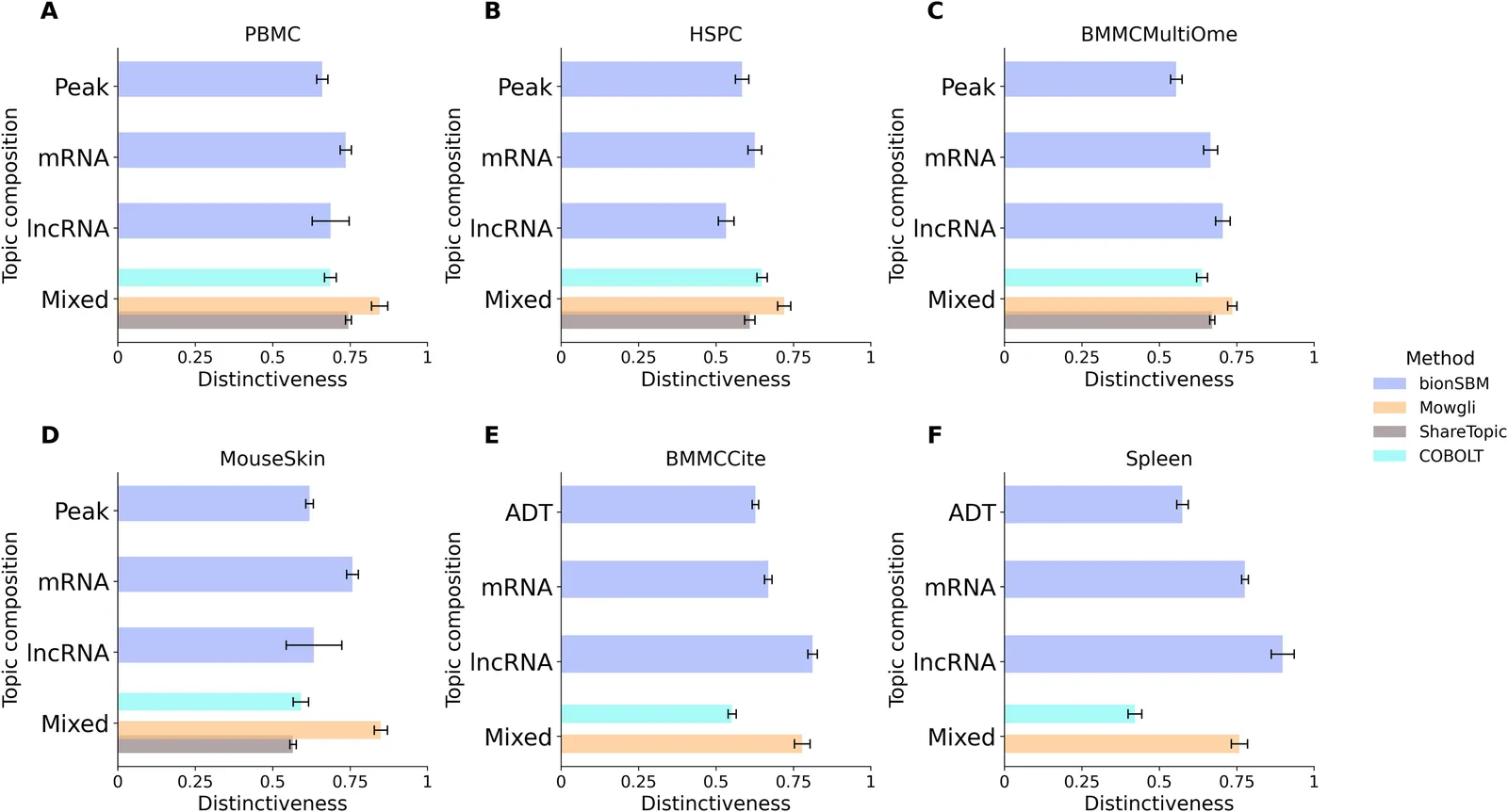
**A**–**F** distinctiveness (a measure of how well topics are separated by quantifying the in-cluster topic probability and the out-of-cluster probability) of the topics for the six data sets. Each bar represents the mean value over multiple runs, and the error bar represents three times the uncertainty on the mean.

Together, these two metrics show that bionSBM’s signatures are highly specific and exhibit strong in-group enrichment relative to out-of-group probabilities; thus, they can be used to assign biological explanations to cell-type groups, making the method easy to interpret and translational. All the methods achieve similar levels of distinctiveness, meaning that all topic models analyzed assign distinct topics per cell type, but bionSBM outperforms the others in specificity, assigning topics to clusters more precisely.

### bionSBM retrieves meaningful chromatin-transcript associations

To assess whether the model learns meaningful chromatin-gene associations, we examined their interplay within and across topics. We quantified the correlation [36] between peak and mRNA topics, as well as the enrichment of transcription factor (TF) motifs and their targets across both modalities, using different approaches (Methods).

Distance correlation (dCor) was computed between mRNA and peak topics to quantify their statistical dependence. For each pair of mRNA and peak topics *(t,u)*, dCor was evaluated using the two corresponding topic vectors across cells, capturing arbitrary dependencies between modalities. This results in a matrix of mRNA–peak topic correlations. The correlation was compared with a null model obtained by independently permuting topic values across cells, thereby preserving marginal distributions (Methods). Final results are reported as Z-scores comparing the observed and null mean dCors (see the Methods section). All data sets show consistent positive Z-scores [Fig. 5A], indicating that the inferred topics encode shared cross-modal structure across cells. This supports the interpretation that chromatin accessibility and transcriptional programs are coordinated at the level of latent topics.

**Figure 5:**
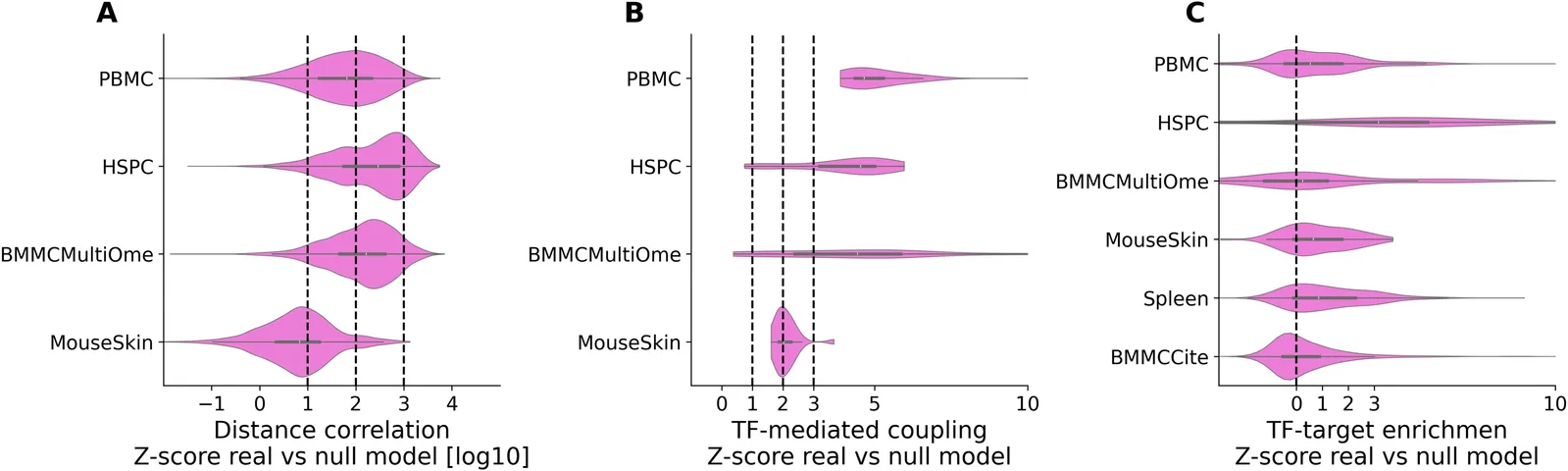
Distance correlation **A**, TF-coupling **B**, and TF-target enrichment **C** for the peak/ADT–mRNA–lncRNA experiment, reported as Z-score between real and permutation-based null model.

To further explore the chromatin-transcriptome interplay, we tested whether mRNA topics are enriched for genes targeted by TFs within peak topics (identified via motif analysis with HOMER [37]). We quantified, for each mRNA–peak topic pair, the enrichment of TF targets within the mRNA-topic gene sets relative to a null model in which the peak-associated TFs were kept fixed and the mRNA gene sets were randomized while matching their size, mean expression, detection coverage, and genomic length (Methods). We obtained an enrichment score matrix for each topic pair and selected the most enriched pairs, assuming that each peak-topic targets primarily one mRNA-topic. We report the results as Z-scores relative to the null model (Methods). The PBMC, HSPC, and BMMCMultiOme data sets show very strong enrichment, whereas the MouseSkin one shows a milder one [Fig. 5B]. Overall, this outcome suggests that bionSBM identifies latent, coordinated ATAC and GEX topics whose gene composition is consistent with known transcriptional regulatory programs.

Finally, for each mRNA topic, we quantified the overlap between its gene set and the set of known TF interactors. We summed, for all TFs present in the topic, the number of shared genes between the topic genes and each TF’s target gene set. We compared this aggregate overlap score against a null distribution obtained from randomly sampled and bias-corrected gene sets as described above (see Methods). All data sets show mildly good levels of TF-enrichment [Fig. 5C], indicating that bionSBM tends to identify mRNA topics structured by known transcriptional regulatory programs. We further investigated the contribution of each modality to the results in Fig. 5 by running the same analysis using combinations of the modalities, in line with the approach used to evaluate cluster NMI/NMI* and topic specificity and distinctiveness. The results show an interplay between chromatin and transcriptome across all the combinations of modalities. However, the lncRNA and the GEX, along with the peaks, tend to show the lowest distribution of dCor, TF-coupling, and TF-enrichment, supporting the argument that it is important to separate different gene types within RNA data to extract the maximum structure from the data [Supplementary Fig. S5 A, B, C]. These results are also consistent with the fold-change metric between the observed values and null models, without normalizing by variance, which shows the magnitude of the observed effect independently of the variance of the null model [Supplementary Fig. S5 D, E, F].

To exemplify the TF–target gene relationship between mRNA and peak topics, we assigned a topic to each cell type using the following criterion: for mRNA and peak modalities, we computed $P_{c}(topic \mid cell\,type)$ and assigned to each cell type the topic with the highest distinctiveness (Methods). We then examined the top-ranked genes by *P(gene ∣ topic)* in the selected mRNA topic and compared them with TF binding motifs enriched in the corresponding peak topic for the same cell type obtained with HOMER [37]. By linking chromatin topics to transcriptome topics, we could extract meaningful case studies that substantiate the interpretability of bionSBM.

This approach recovered well-established regulatory programs across data sets. In PBMCs, the peak-topic with the highest distinctiveness for the B memory cell population was enriched for the PAX5 motif [Fig. 6A], an essential gene required for B cell commitment from lymphoid progenitors [38]. Coherently, the same topic was also the highest enriched in B naive cells [Supplementary Fig. S6 A], and the same gene was found to be in the most enriched mRNA-topic in both cell states [Fig. 6A, Supplementary Fig. S6 A]. Notably, the topics were highly enriched for both mRNA and ATAC features only in B cell populations [Supplementary Fig. S7 A], highlighting the specificity of our approach. In the HSPC data set, granulocyte-monocyte progenitors (GMPs) showed the strongest association with a peak-topic motif for IRF8 [Fig. 6B], a TF known to regulate GMP differentiation [39]. The corresponding highest-associated mRNA-topic for the same cell type contained the same TF [Fig. 6B], again connecting the two feature spaces mechanistically. Lastly, in the BMMCMultiOme data set, the proerythroblasts, normoblasts, and erythroblasts had a high probability of a peak-topic showing the KLF1 motif [Fig. 6C, Supplementary Fig. S6 B, Supplementary Fig. S6 C], a master regulator of erythropoiesis [40], and the same gene belonged to the most relevant mRNA-topic for the three above-mentioned cell types, which are the primary cell states evolving during erythropoiesis [Fig. 6C, Supplementary Fig. S6 B, Supplementary Fig. S6 C]. Again, the selected mRNA- and peak-topics were not only the ones with the highest distinctiveness among all other topics, but they were also highly enriched only for the cell types under examination [Supplementary Fig. S7], giving evidence that the explanations are specific. We next evaluated the association of the selected mRNA and peak topics with the bionSBM clusters using $P_{c}(\mathrm{topic}\mid \mathrm{cluster})$. The cluster-enrichment step is label-independent but is applied to topics previously selected using the annotated cell types [Fig. 6]. Specifically, for each selected topic, we identified the bionSBM clusters showing the strongest enrichment without using cell-type labels. We then examined the cell-type composition of these clusters as a *post hoc* validation step. For the selected topics, the most strongly enriched clusters were predominantly composed of the expected cell types, supporting the biological consistency between the inferred cell partitions and the regulatory programs captured by the selected topics [Supplementary Fig. S8].

**Figure 6:**
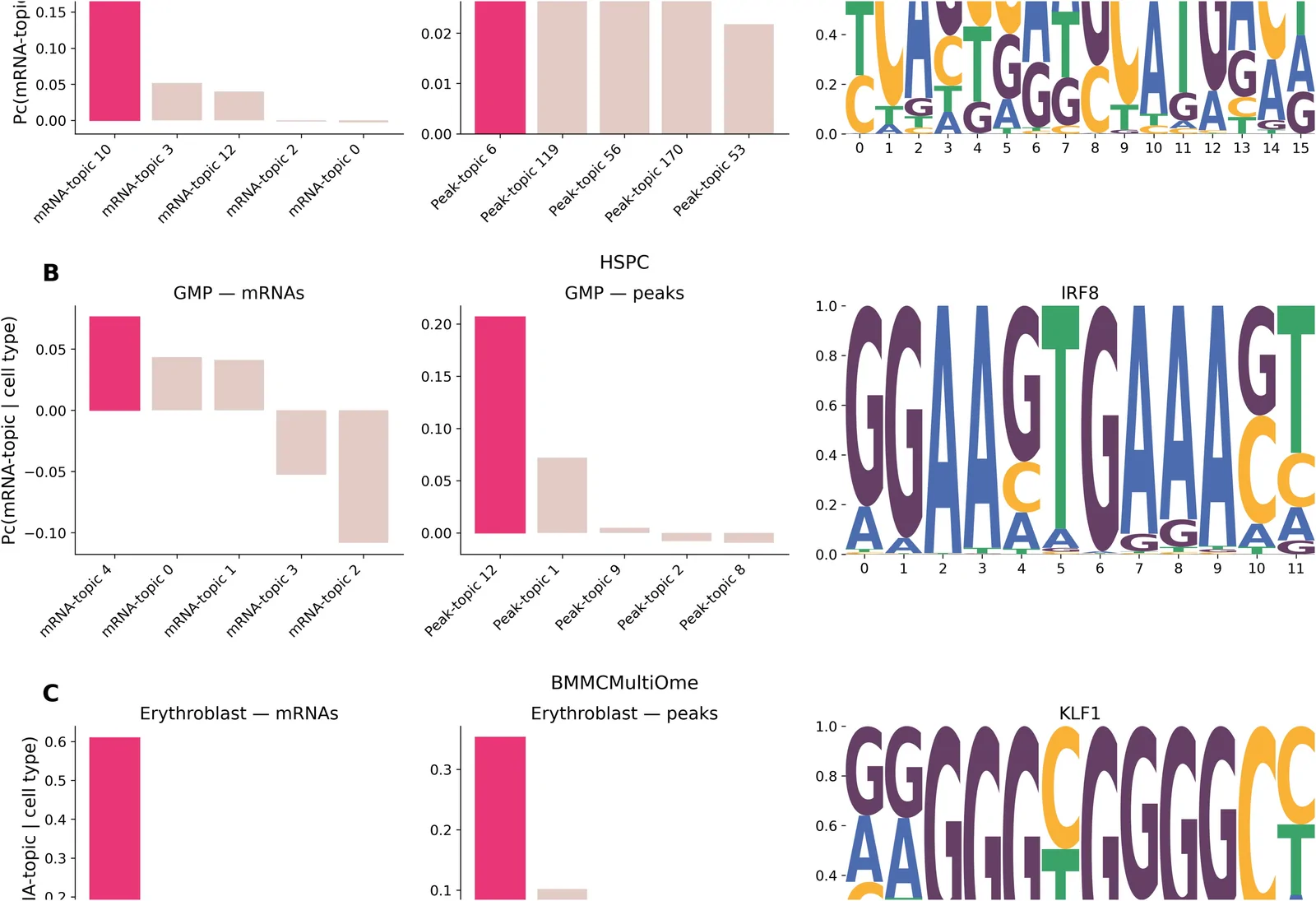
We present a row for each dataset. **Left**: enrichment profile of the mRNA-topic with the highest distinctiveness for the given cell type. **Middle**: enrichment profile of the peak-topic with the highest distinctiveness for the given cell type. **Right**: sequence logo of a significantly enriched TF motif identified in peaks belonging to the selected peak-topic (HOMER analysis).

Overall, the discovered peak and mRNA assignments consistently recovered canonical lineage-defining TFs, supporting the bionSBM integrative topic model’s ability to capture biologically meaningful, cell-type-specific regulatory programs across modalities and to link the transcriptome to the epigenome.

## Discussion

In this study, we present bionSBM, a graph-based SBM to integrate single-cell multi-omic data [Fig. 1]. This approach has both theoretical and practical advantages over the state-of-the-art topic models for the single-cell field. First, our method can be applied to any multi-modal data, since it handles an arbitrary number of partitions (i.e. modalities). Second, the method automatically detects the optimal number of topics and clusters without requiring these values to be specified as hyperparameters. Moreover, bionSBM treats modalities symmetrically *a priori* and does not introduce modality-specific weighting parameters. Finally, because topic modelling algorithms are Bayesian, they require specification of a prior distribution over topics. Rather than imposing a structured distribution that constrains cells and features to follow a single mixture model, we employ a flat (non-informative) prior.

Integrating multiple omics layers is key to uncovering new biological insights. By modelling separate topic compositions for each network layer, bionSBM captures coherent relationships across modalities, thereby inferring modality-specific topic structures within a unified probabilistic framework and enabling independent yet coherent biological interpretations across omics layers.

bionSBM also improves performance in cell-type retrieval, outperforming state-of-the-art approaches and more accurately recovering known biological annotations [Fig. 2]. These performance gains come along with inherent interpretability. The model generates specific [Fig. 3], distinct [Fig. 4], and stable [Supplementary Fig. S9, Supplementary Fig. S10] topic representations. These representations support cross-modal regulatory analyses [Fig. 5, Supplementary Fig. S5] and provide biologically meaningful explanations of annotated cell groups and clusters [Fig. 6, Supplementary Fig. S6, Supplementary Fig. S7], Supplementary Fig. S8. This balance between accuracy and interpretability is essential, as predictive improvements alone are unlikely to drive meaningful biological discovery.

We released a Python implementation of the tool, integrated into the single-cell universe, leveraging the convenient *AnnData* and *Muon* packages to guarantee reproducibility and broad usability of the method. Here, we have applied bionSBM to single-cell multi-omic measurements with scRNA-seq, paired with either scATAC-seq or surface protein measurements; however, the framework can be used for any new omics measurements that can be summarized as a count matrix, making the approach truly generalizable.

## Methods

### Single-cell data processing

The raw reads of the PBMC [32] data set were processed using *Cell Ranger ARC 2.0.2* [41], aligning them to the complete human genome (T2T) [42]. The gene expression count matrices for all other data sets (BMMCMultiOme [33], BMMCCite [35], HSPC [34], MouseSkin [4], Spleen [28]) were obtained from the literature, along with the cell type annotations. From the total GEX count matrices, which contain all the families of transcripts that scRNA-seq can profile, we extracted the mRNA and lncRNA counts using the genome annotations from the appropriate GTF file for the data set (see Supplementary Table S1 for the files). All matrices underwent the same preprocessing according to standard single-cell best practices [6] using *Scanpy* [43]: quality control, doublet identification [44], filtering, library-size normalization, and log transformation. The PBMC peak count matrix was built with Episcanpy [45] after MACS2 [46] peak calling. All the other peak count matrices were obtained from the literature. Similar to scRNA-seq data, preprocessing for scATAC-seq followed best practices, including quality control, filtering, and library size normalization. In both scRNA-seq and scATAC-seq matrices, we kept only the highly variable features. The ADT (surface protein) count matrices were library-size normalized only because the number of features is minimal, so no additional filters are needed.

### Stochastic block models

The statistical inference procedure and the definitions of topics and probability distributions are based on the hSBM algorithm. The input matrices are transformed into a weighted multipartite network. Each node represents a feature or a cell (i.e. mRNA, lncRNA, peak ADT). To build the network, we use the log-counts of the data with standard preprocessing, as discussed in the previous paragraph. Each cell is connected to a feature, in one of the network partitions, if that feature is represented by a non-zero entry in the count matrix; the edge is weighted by the count itself. In the comparison of bionSBM to other algorithms, focusing on the differences, advantages, and drawbacks among algorithms, we used this single strategy to build the bionSBM network, rather than optimizing preprocessing for bionSBM. The algorithm defines *P(model ∣ data)*, where the data are the graph and the model is the particular partition constructed during inference. The inference process begins with nodes (i.e. genes or cells) assigned to random blocks (either clusters or topics). The process consists of a Markov Chain Monte Carlo (MCMC) simulation to adjust node assignments to blocks, thereby minimizing the DL [47], defined in natural units as the number of bits required to encode the model and the data. Moves in the MCMC are accepted with probability [21]:


(1) \begin{equation*} P\left(r\to s|t\right) = \frac{e_{ts} + \epsilon}{e_{t} + \epsilon B},\end{equation*}

which represents the probability of moving a node from block (cluster or topic) *r* to block *s* if a random neighbour of the considered node belongs to block *t*; given that *B* is the total number of blocks, $e_{ts}$ is the number of edges from group *t* to group *s* and $e_{t}$ is the total number of edges within group *t*. Such probability is proportional to the ratio between the number of edges that connect group *t* and block *s* and the number of edges that go to block *t* to group *s* and $e_{t}$ is the total number of edges within group *t*. Such probability is proportional to the ratio between the number of edges that connect group *t* and block *s* and the number of edges that go to block *s*, plus a term that is necessary in order to have a uniform probability if there are no connections between the blocks considered. For instance, if all the edges from *t* go to group *s*, $e_{ts}$ equals $e_{t}$ and the probability becomes uniform: *1/B*. The algorithm supports multiple random initializations, i.e. different starting points for estimating the posterior probability.

It is essential to highlight that, given the probability of accepting a move, the advantage of using multibranch SBM on multi-omics problems is clear. When moving a cell from block *r* to block *s*, the algorithm samples a neighbour of the cell and it labels its group as *t*. At this point, the model only considers the links between the cell and the nodes in the group, so the normalizations of other branches are irrelevant. The final output of the model consists of a hierarchy of topics and clusters. For each level in the hierarchy, the model returns the grouping of features into topics, the grouping of cells into clusters, and the probability that each cell belongs to each topic [18, 19, 22]. When the data set annotation is not available, we suggest that the user excludes partitions where the number of clusters or topics is either one or is disproportionately high with respect to the expected heterogeneity of the data, and investigates the heterogeneity of the data using all remaining levels to perform a multi-scale analysis.

A first implementation of multipartite hSBM, the so-called nSBM, is available at https://github.com/BioPhys-Turin/nsbm [22] based on the graph-tool library [48].

### Experiment running and metrics

bionSBM has been run 25 times for each combination of feature spaces, with the initial seed changed each time and the number of initializations set to 7 as suggested in [23]. The ShareTopic, Mowgli, COBOLT, MultiVI, and totalVI experiments have also been run 25 times. MultiVI and totalVI were run using the *scvi-tools* framework [49]. All the algorithms have been run with default parameters, with the number of topics (or factors, or latent dimensions) equal to the number of cell types annotated in each data set. In Mowgli, we set the regularization strength to 0.05 across all feature spaces to treat them equally; 0.05 comes from the average suggested weight in their code between RNA and ATAC data. For each experiment outcome and each level of the bionSBM hierarchy, we have measured the NMI/NMI* between the algorithms’ clustering and the original cell-type annotation.

The NMI/NMI* is a version of the normalized mutual information (NMI) [50] that accounts for randomness, with NMI* quantifying the agreement between the ground-truth label and a random clustering of cells. In this way, we account for partitions with a large number of clusters that would otherwise yield a falsely high NMI. On a log scale, the lower limit is 0, indicating that the clustering is entirely dominated by randomness; i.e. the NMI equals NMI*. For the comparator methods, the number of topics/factors/latent dimensions was set to the number of annotated cell types for benchmarking. bionSBM inference itself does not use the annotations. After inference, for each run and experiment, we used the annotations only to identify the hierarchy level with the highest NMI/NMI*. The resulting scores were then averaged across runs for each method–feature-space combination to produce Fig. 2.

### Topics specificity, distinctiveness, and stability

To compute the specificity, distinctiveness, and stability we selected, for each experiment and for each run, the level with the closest number of clusters to the ground truth annotation. After setting the level, we assigned to each group of cells the topic with the highest probability. Let *t* and *i* be a topic and a cell, respectively, we used a modified version of the *P(t|i)* defined as $P_{c}(t|i)$ (where c stands for centred) to exclude topics that are important for all the cells, which would be the equivalent of the topic of articles and prepositions in NLP, as suggested in [18]. The centred probability is defined as


(2) \begin{align*}& P_{c}(t \mid i) = P(t \mid i) - \frac{1}{|\mathcal{C}|} \sum_{j \in \mathcal{C}} P(t \mid j),\end{align*}

where $\mathcal{C}$ denotes the set of all cells. Let $\mathcal{C}_\tau $ be the set of cells belonging to a particular group *τ *, we can derive the $P_{c}(t \mid \tau )$ as


(3) \begin{align*}& P_{c}(t \mid \tau) = \frac{1}{|\mathcal{C}_\tau|} \sum_{i \in \mathcal{C}_\tau} P_{c}(t \mid i)\end{align*}

We assigned a topic to each cell group according to $t^{*}_\tau = \underset{t}{\arg \max }\; P_{\mathrm{c}}(t \mid \tau )$. We then counted the number of distinct topics assigned to the cell groups and divided that number by the total number of cell groups: let $N_{\mathrm{ct}}$ be the total number of cell types and let $N_{\mathrm{assigned}}$ be the number of distinct dominant topics assigned across the $N_{\mathrm{ct}}$ cell types, the specificity is then defined as


(4) \begin{align*}& \mathrm{Specificity} = \frac{N_{\mathrm{assigned}}}{N_{\mathrm{ct}}}\end{align*}

We select the hierarchy level in bionSBM with the closest number of topics to Mowgli and ShareTopic. We average the values obtained from the tool runs to produce the bar plot in Fig. 3.

Once we marked the most important topic for each group as described above, we quantified the distinctiveness of these assigned topics, which is defined as


(5) \begin{align*}& \mathrm{Distinctiveness}(t_\tau^{*}) = \frac{1}{|\mathcal{C}_\tau|} \sum_{i \in \mathcal{C}_\tau} P_{c}(t_\tau^{*} \mid i) - \frac{1}{|\mathcal{C} \setminus \mathcal{C}_\tau|} \sum_{i \in \mathcal{C} \setminus \mathcal{C}_\tau} P_{c}(t_\tau^{*} \mid i),\end{align*}

where $t_\tau ^{*}$ is the topic assigned to group $\mathcal{C}_\tau $, and $P_{c}(t|i)$ is defined as in 2. In other words, it is defined as the difference between the average probability that the topic belongs to the cells within the group and the average probability that it belongs to cells outside the group. For distinctiveness, we average the values across multiple model runs to produce Fig. 4.

To quantify the stability of topic–feature associations across independent runs of the same experiment, we measured the similarity of the top three most important topics identified for each cell type. For a given run *r*, let $P_{c,r}(t \mid i)$ denote the centred posterior probability of topic *t* for cell *i*, as obtained from the topic–document matrix as shown in Eq 2.

For each cell type *τ *, a cell-type-specific topic score was computed by averaging centred probabilities across cells of that type:


(6) \begin{align*}& P_{c,r}(t \mid \tau) = \frac{1}{|\mathcal{C}_\tau|} \sum_{i \in \mathcal{C}_\tau} P_{c,r}(t \mid i),\end{align*}

where $\mathcal{C}_\tau \subset \mathcal{C}$ is the subset of cells annotated as cell type *τ *. For each run and cell type, the set $\mathcal{T}_{r}^\tau $ of the top $N_{\mathrm{top}}=3$ topics with the highest values of $P_{c,r}(t \mid \tau )$ was selected. Each topic $t \in \mathcal{T}_{r}^\tau $ is associated with a topic–feature probability vector $\boldsymbol{\phi }_{r,t}$.

To compare two runs *r* and $r^{\prime}$, we computed pairwise similarities between all topic vectors in $\mathcal{T}_{r}^\tau \times \mathcal{T}_{r^{\prime}}^\tau $ using the cosine similarity


(7) \begin{align*}& S_{\mathrm{cos}}(p, q) = \frac{p \cdot q}{\|p\| \, \|q\|},\end{align*}

where *p* and *q* are normalized topics–features distributions.

For each cell type and run pair, the similarity was defined as the maximum similarity across possible combinations given the three top topics for run pairs:


(8) \begin{align*}& S_{\mathrm{stab}}^\tau(r,r^{\prime}) = \max_{\substack{ t \in \mathcal{T}_{r}^\tau \\ u \in \mathcal{T}_{r^{\prime}}^\tau } } S_{\mathrm{cos}}\!\left( \boldsymbol{\phi}_{r,t}, \boldsymbol{\phi}_{r^{\prime},u} \right)\end{align*}

Choosing the three top topics makes the metric robust to slight variations in the top rankings, which arise from data noise and the approximation introduced by the sampling procedure. The final stability score for an experiment was obtained by averaging $S_{\mathrm{stab}}^\tau (r,r^{\prime})$ across all cell types and all unordered pairs of runs.

### Topics analysis and interpretation

For biological interpretation, we used the reference cell-type annotations to identify topics with the highest mean loading in each annotated population. We then assessed the motif and regulon content of these topics. This analysis evaluates concordance between inferred topics and established cell-type regulatory programs; it is not intended as a label-independent discovery analysis. For the TF-based analyses, random mRNA gene sets were matched to the observed topic gene sets in size, mean expression, detection coverage, and genomic gene length. Mean expression and coverage were computed from the expression matrices, with coverage defined as the fraction of cells with non-zero expression. Gene lengths were obtained from the appropriate GTF files (Supplementary Table S1). To account for these covariates, we divided genes into up to five quantile bins for each variable (expression, coverage, and length) and grouped them by their combinations of bins. Random gene sets were then sampled without replacement, preserving the number of observed topic genes within each stratum. Observed topic genes were excluded from the sampling pool whenever sufficient alternative genes were available. No random peak gene sets were constructed.

To quantify statistical dependence between RNA and chromatin latent representations, we consider two matrices $X \in \mathbb{R}^{N \times K_{x}}$ and $Y \in \mathbb{R}^{N \times K_{y}}$, where rows correspond to cells and columns to topics and each topic is mean-centred across cells. For each pair of topics *(t,u)*, we compute the dCor, which captures arbitrary statistical dependence. Let $x_{t} \in \mathbb{R}^{N}$ and $y_{u} \in \mathbb{R}^{N}$ denote the *t*th and *u*th topic vectors across cells. We first construct pairwise distance matrices:


(9) \begin{align*}& D^{x}_{ij} = |x_{t}^{(i)} - x_{t}^{(j)}| \quad \mathrm{and} \quad D^{y}_{ij} = |y_{u}^{(i)} - y_{u}^{(j)}|\end{align*}

for all cell pairs *(i,j)*. These matrices are then double-centred to remove mean effects:


(10) \begin{align*} & A_{ij} = D^{x}_{ij} - \bar{D}^{x}_{i\cdot} - \bar{D}^{x}_{\cdot j} + \bar{D}^{x}_{\cdot\cdot} \end{align*}

(11) \begin{align*} & B_{ij} = D^{y}_{ij} - \bar{D}^{y}_{i\cdot} - \bar{D}^{y}_{\cdot j} + \bar{D}^{y}_{\cdot\cdot}, \end{align*}

where $\bar{D}_{i\cdot }$, $\bar{D}_{\cdot j}$, and $\bar{D}_{\cdot \cdot }$ denote row, column, and global means, respectively. Distance covariance and variance are defined as


(12) \begin{align*} & \mathrm{dCov}(x_{t}, y_{u}) = \mathbb{E}[A_{ij} B_{ij}] \end{align*}

(13) \begin{align*} & \mathrm{dVar}(x_{t}) = \mathbb{E}[A_{ij}^{2}] \quad \mathrm{and} \quad \mathrm{dVar}(y_{u}) = \mathbb{E}[B_{ij}^{2}]\end{align*}

and dCor is given by


(14) \begin{align*}& \mathrm{dCor}(x_{t}, y_{u}) = \frac{ \mathrm{dCov}(x_{t}, y_{u}) } { \sqrt{ \mathrm{dVar}(x_{t}) \mathrm{dVar}(y_{u}) } }\end{align*}

This procedure yields a dependence matrix $\mathbf{C} \in \mathbb{R}^{K_{x} \times K_{y}}$ with entries $C_{tu} = \mathrm{dCor}(x_{t}, y_{u})$. For computational efficiency, dCor is evaluated on a random subset of 3000 cells. We then constructed a null model by independently permuting each topic across cells:


(15) \begin{align*}& x_{t} \rightarrow \pi^{x}_{t,m}(x_{t}), \quad y_{u} \rightarrow \pi^{y}_{u,m}(y_{u}),\end{align*}

which preserves marginal distributions. The same procedure is applied to the permuted data over 20 replicates, yielding a set of null modelling correlation matrices $\{\widehat{\mathbf{C}}^{(m)}\}_{m=1}^{20}$, which averages as $\mu _{tu}$ with SD $\sigma _{tu}$. We then defined a Z-score


(16) \begin{align*}& z^{\mathrm{dCor}}_{tu} = \frac{ C_{tu} - \mu_{tu} } { \sigma_{tu} },\end{align*}

which provides a normalized measure of how strongly RNA and chromatin topic structures are statistically coupled beyond chance. The same procedure is repeated for all 25 bionSBM runs. The resulting data are plotted in Fig. 5A.

To quantify the regulatory consistency between chromatin accessibility and gene expression topics inferred by bionSBM, we evaluated the enrichment of TF regulons within gene sets. Let $\mathbf{Z} \in \mathbb{R}^{|\mathcal{G}| \times K}$ denote the gene–topic matrix for GEX, where $\mathcal{G}$ is the set of genes and *K* the number of topics. For each topic *t*, we define the corresponding gene set $\mathcal{G}_{t} = \{ g \in \mathcal{G} \mid Z_{gt}> 0 \}$. For each ATAC topic *a*, we extract a set of TFs $\mathcal{F}_{a}$ based on significantly enriched motifs (Benjamini–Hochberg FDR [51] *<0.05* from HOMER analysis). Using a prior TF–target network (CollecTRI [52, 53]), where each TF *f* is associated with a regulon $R_{f} \subseteq \mathcal{G}$, we compute a coupling score:


(17) \begin{align*}& S_{\mathrm{coup}}(\mathcal{G}_{t}, \mathcal{F}_{a}) = \sum_{f \in \mathcal{F}_{a}} \frac{ |\mathcal{G}_{t} \cap R_{f}| } { |R_{f}| }\end{align*}

This score measures the fraction of each TF regulon captured by the gene set, normalized by regulon size. The null model was computed over $N_{\mathrm{perm}}^{\mathrm{coup}} = 1000$ permutations. From this null distribution, we estimate the mean $\mu ^{\mathrm{coup}}_{ta}$ and standard deviation $\sigma ^{\mathrm{coup}}_{ta}$, and compute a Z-score:


(18) \begin{align*}& z^{\mathrm{coup}}_{ta} = \frac{ S_{\mathrm{coup}}(\mathcal{G}_{t}, \mathcal{F}_{a}) - \mu^{\mathrm{coup}}_{ta} } { \sigma^{\mathrm{coup}}_{ta} }\end{align*}

This procedure is repeated for all ATAC–GEX topic pairs across $N_{\mathrm{runs}} = 25$ independent runs, yielding a matrix of regulatory coupling scores. For both observed and randomized data, coupling scores were calculated for all peak–mRNA topic pairs, ranked, and the maximum score retained. The observed maximum was then compared with the distribution of maximum scores obtained from the randomized data. The resulting data are plotted in Fig. 5B.

To assess whether the gene sets associated with each mRNA-topic capture biologically meaningful regulatory structure, we performed a TF enrichment analysis using curated regulons. For each topic *t*, where $\mathcal{G}$ denotes the set of all genes, we defined a global overlap score


(19) \begin{align*}& S_{\mathrm{enr}}(\mathcal{G}_{t}) = \sum_{f} |\mathcal{G}_{t} \cap R_{f}|,\end{align*}

where $R_{f}$ is the target set of TF *f*. To evaluate the significance of this score, we constructed a null distribution by sampling random gene sets $\mathcal{G}_{t}^{(m)}$ of the same size, coverage, expression, and gene length as $\mathcal{G}_{t}$ from $\mathcal{G}$ and computing $S_{\mathrm{enr}}(\mathcal{G}_{t}^{(m)})$ over $N_{\mathrm{perm}}^{\mathrm{enr}}=1000$ permutations. The mean and standard deviation of the null distribution were estimated as


(20) \begin{align*}& \mu^{\mathrm{enr}}_{t,\mathrm{null}} = \frac{1}{N_{\mathrm{perm}}^{\mathrm{enr}}} \sum_{m=1}^{N_{\mathrm{perm}}^{\mathrm{enr}}} S_{\mathrm{enr}}(\mathcal{G}_{t}^{(m)})\end{align*}

and


(21) \begin{align*}& \sigma^{\mathrm{enr}}_{t,\mathrm{null}} = \sqrt{ \frac{1}{ N_{\mathrm{perm}}^{\mathrm{enr}}-1 } \sum_{m=1}^{N_{\mathrm{perm}}^{\mathrm{enr}}} \left[ S_{\mathrm{enr}}(\mathcal{G}_{t}^{(m)}) - \mu^{\mathrm{enr}}_{t,\mathrm{null}} \right]^{2} }.\end{align*}

We then defined the enrichment Z-score as


(22) \begin{align*}& z^{\mathrm{enr}}_{t} = \frac{ S_{\mathrm{enr}}(\mathcal{G}_{t}) - \mu^{\mathrm{enr}}_{t,\mathrm{null}} } { \sigma^{\mathrm{enr}}_{t,\mathrm{null}} },\end{align*}

which quantifies the deviation of the observed TF–target overlap from the permutation-based null expectation in units of the null standard deviation. The resulting Z-scores are plotted in Fig. 5C.

For all three metrics described in this chapter, we also computed the ratio between the real and null model means, namely


(23) \begin{align*}& R_{M} = \frac{ \mu^{M}_{\mathrm{real}} } { \mu^{M}_{\mathrm{null}} }, \qquad M \in \{ \mathrm{dCor}, \mathrm{coup}, \mathrm{enr} \}\end{align*}

to produce Supplementary Fig. S5 D–F.

**Key Points**

- We introduce a probabilistic framework based on multipartite graphs for the joint analysis of single-cell multi-omic data.
- The model enables unified representation of heterogeneous modalities, identifying shared latent biological topics.
- It captures regulatory structure by linking genes, peaks, and surface proteins within a single generative framework.
- The approach shows strong interpretability and competitive performance compared with existing integration methods.
- The framework provides a strategy to uncover coordinated molecular programs in complex datasets.

## Supplementary Material

bionSBM_supplementary_v1_bbag608

## Data Availability

Code and tutorial for bionSBM are available at https://github.com/gmalagol10/bionsbm.git. The code to reproduce all the results is available at https://github.com/gmalagol10/bionsbm/tree/main/reproducibility.
