# Supplementary material for: Integration of single-cell multi-omic data with graph-based topic modelling
Source: Brief Bioinform. 2026 Nov 15;27(6):bbag608. doi: 10.1093/bib/bbag608 (PMC13770250; doi:10.1093/bib/bbag608)
Supplement: Supplementary file 1 [file bionsbm_supplementary_v1_bbag608.pdf]

Additional file 1: Supplementary tables

	Number of cell types	Number of peaks or surface molecules	Number of features in GEX	Number of protein-coding genes	Number of lncRNAs	GTF file source
BMMCCite	37	134	3782	3977	589	https://www.gencodegenes.org/human/release_44.html
BMMCMultiOme	21	31667	3622	1258	486	https://www.gencodegenes.org/human/release_44.html
HSPC	11	20400	5377	5553	2242	https://www.gencodegenes.org/human/release_44.html
MouseSkin	23	31114	4425	5131	2648	https://www.gencodegenes.org/mouse/release_M33.html
PBMC	12	19125	4141	2792	2330	https://github.com/marbl/CHM13
Spleen	35	209	5067	3954	798	https://www.gencodegenes.org/mouse/release_M33.html

Table S1: Metadata and properties of the data sets considered in this study.

Additional file 2: Supplementary figures

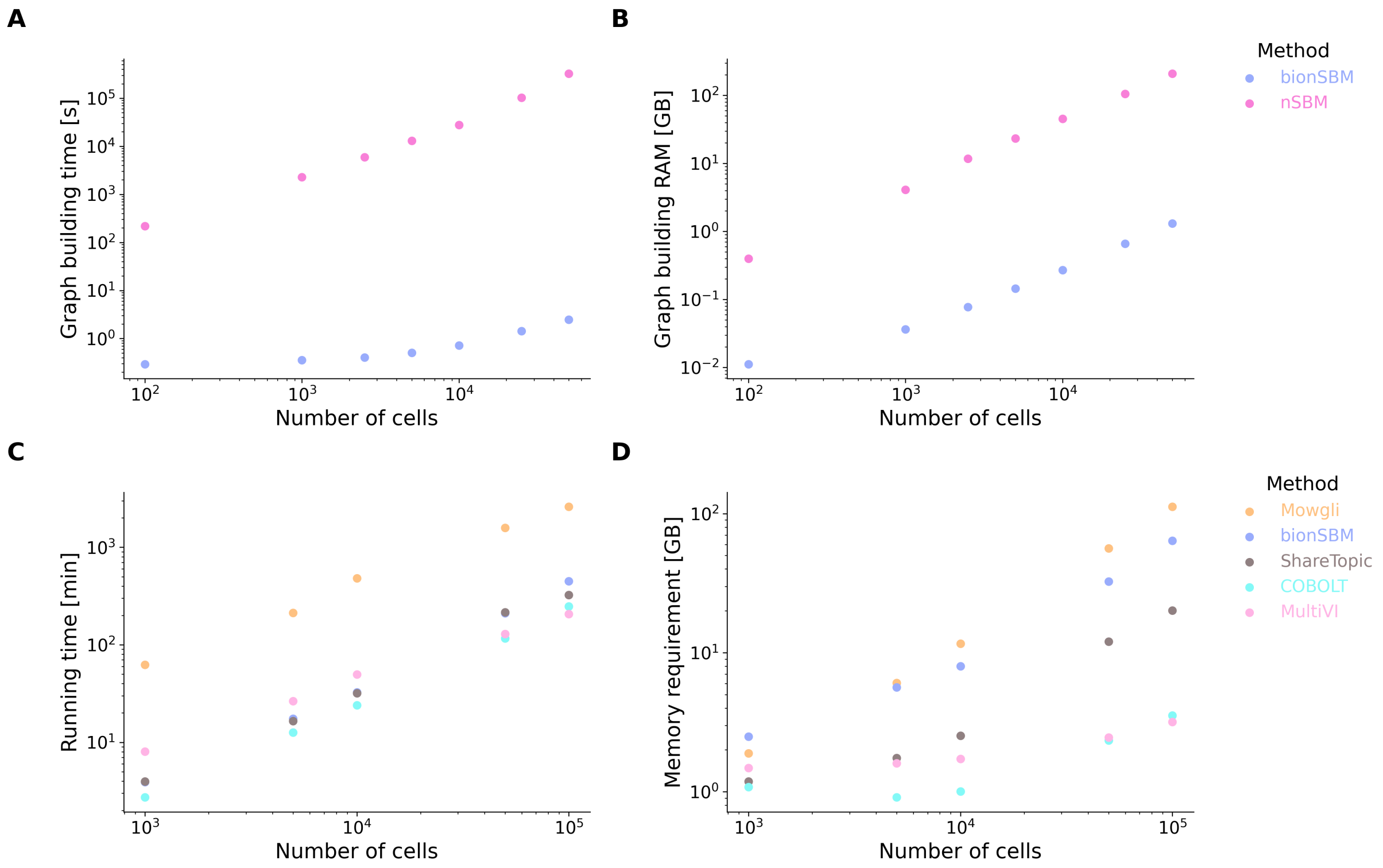

Fig. S1: **A**–**B** Performance varying the number of cells of bionSBM and nSBM in creating the graph from the count matrix. The downsampling is stratified by cell-type abundance to preserve the biological structure of the data set. The running time (**C**) and memory requirements (**D**) of the methods varying with the number of cells.

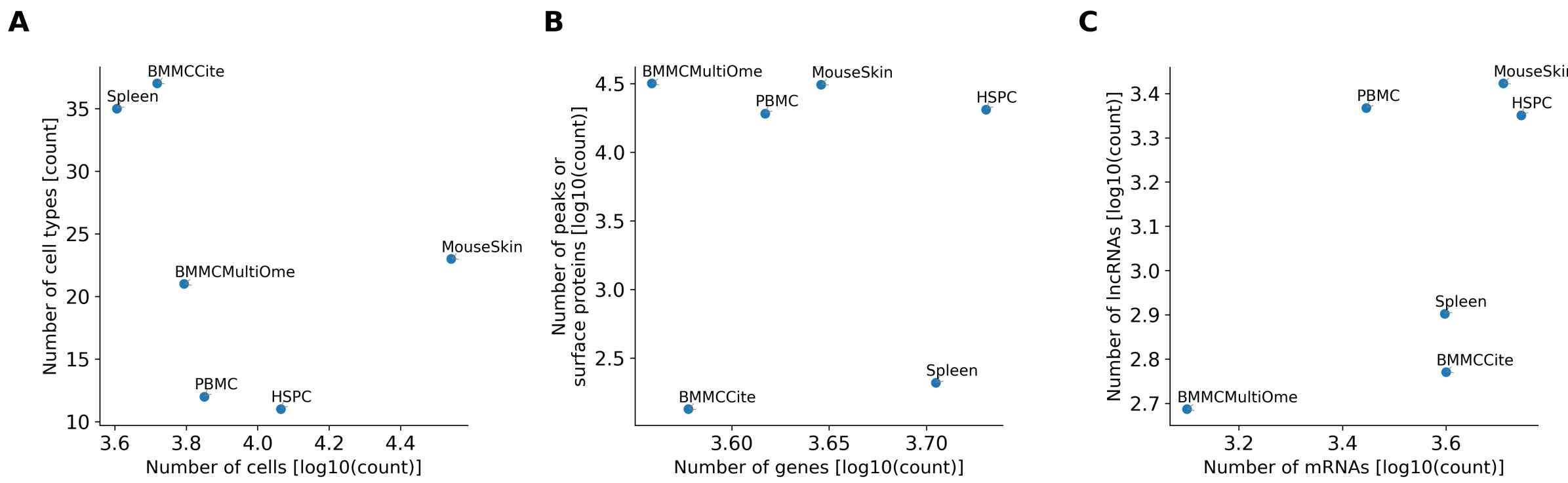

Fig. S2: The complexity of the six data sets measured by the number of cells, cell types and features.

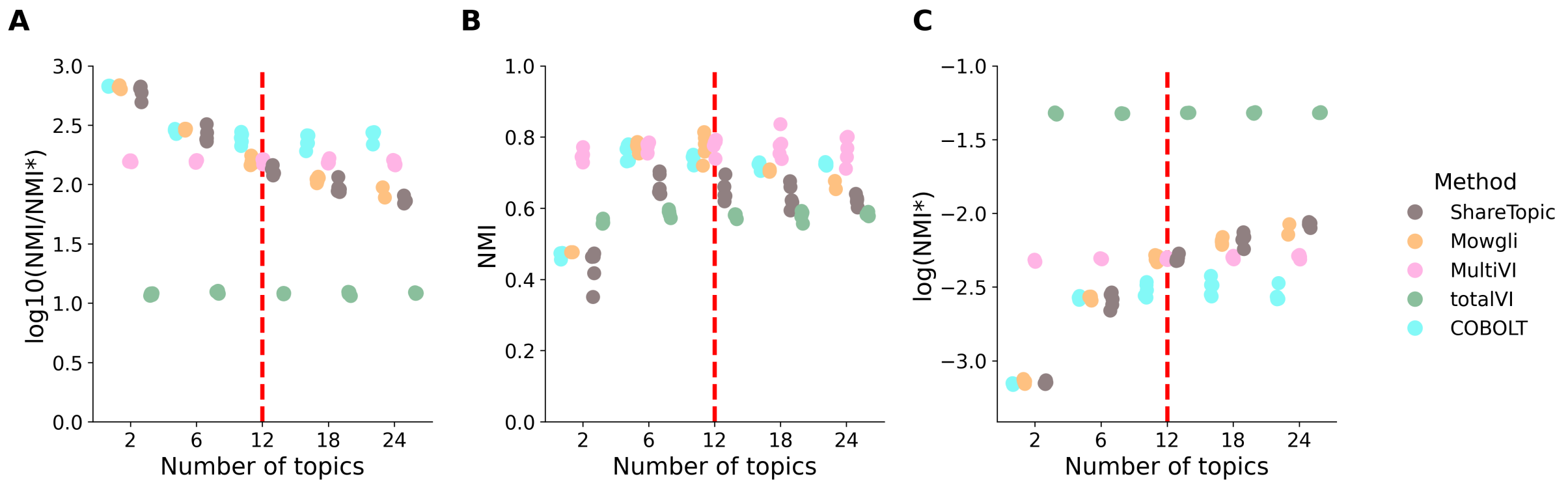

Fig. S3: Trend of NMI/NMI* (**A**), NMI (**B**), and NMI* (**C**) for the comparator methods as the number of topics/factors/latent dimensions is varied for the PBMC data set.

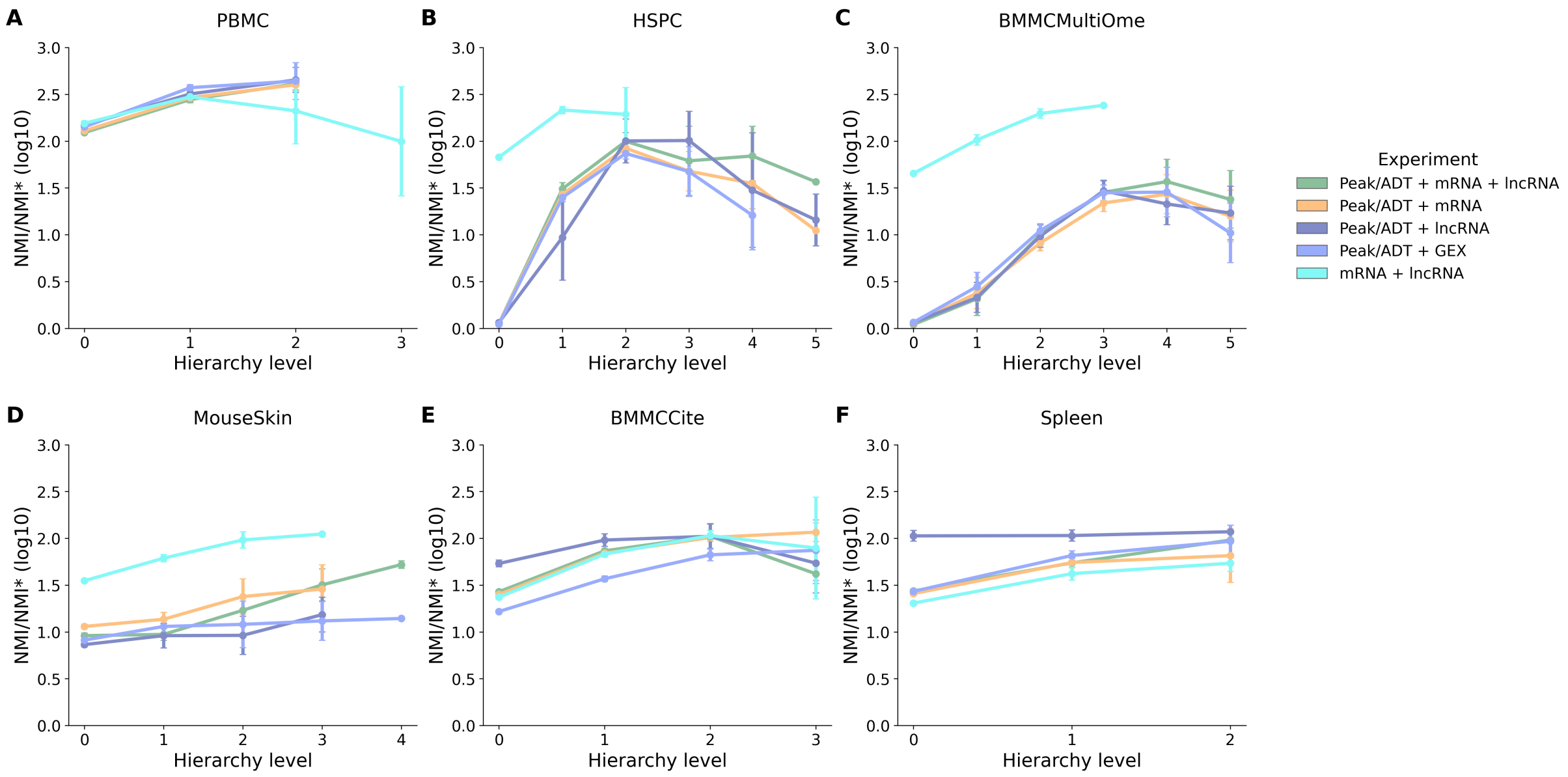

Fig. S4: NMI/NMI* across all inferred hierarchy levels of bionSBM for the six datasets. Colours indicate the different modality combinations. Points represent the mean log10(NMI/NMI*) across 25 independent runs, and error bars represent three times the standard error of the mean.

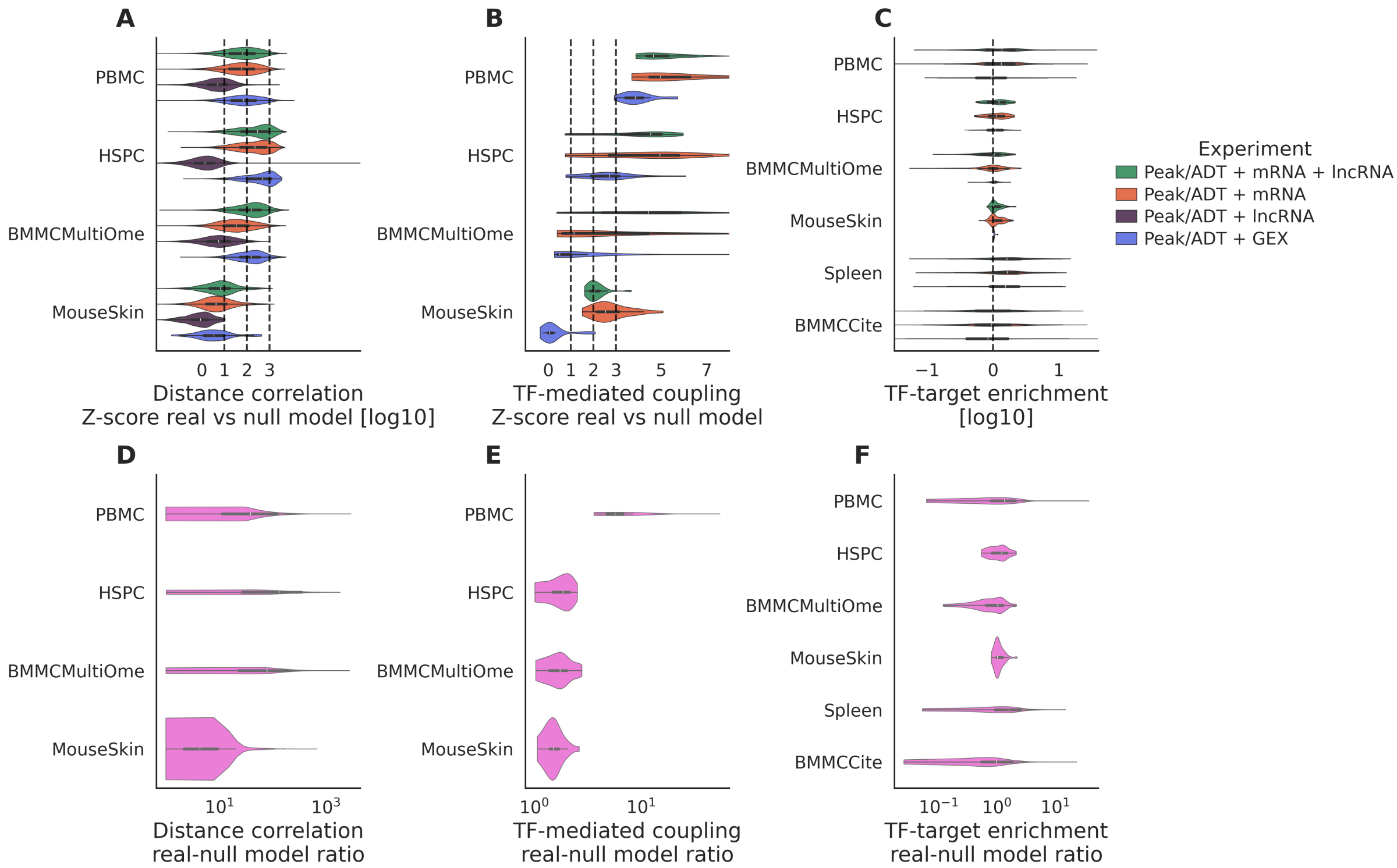

Fig. S5: Distance correlation (**A**), TF-coupling (**B**) and TF-target enrichment (**C**) stratified by a combination of omics to show the contribution of each molecular layer reported as Z-score between real and null model. Distance correlation (**D**), TF-coupling (**E**), and TF-target enrichment (**F**) for the peak/ADT-mRNA-lncRNA experiment, reported as ratios between the real and null models.

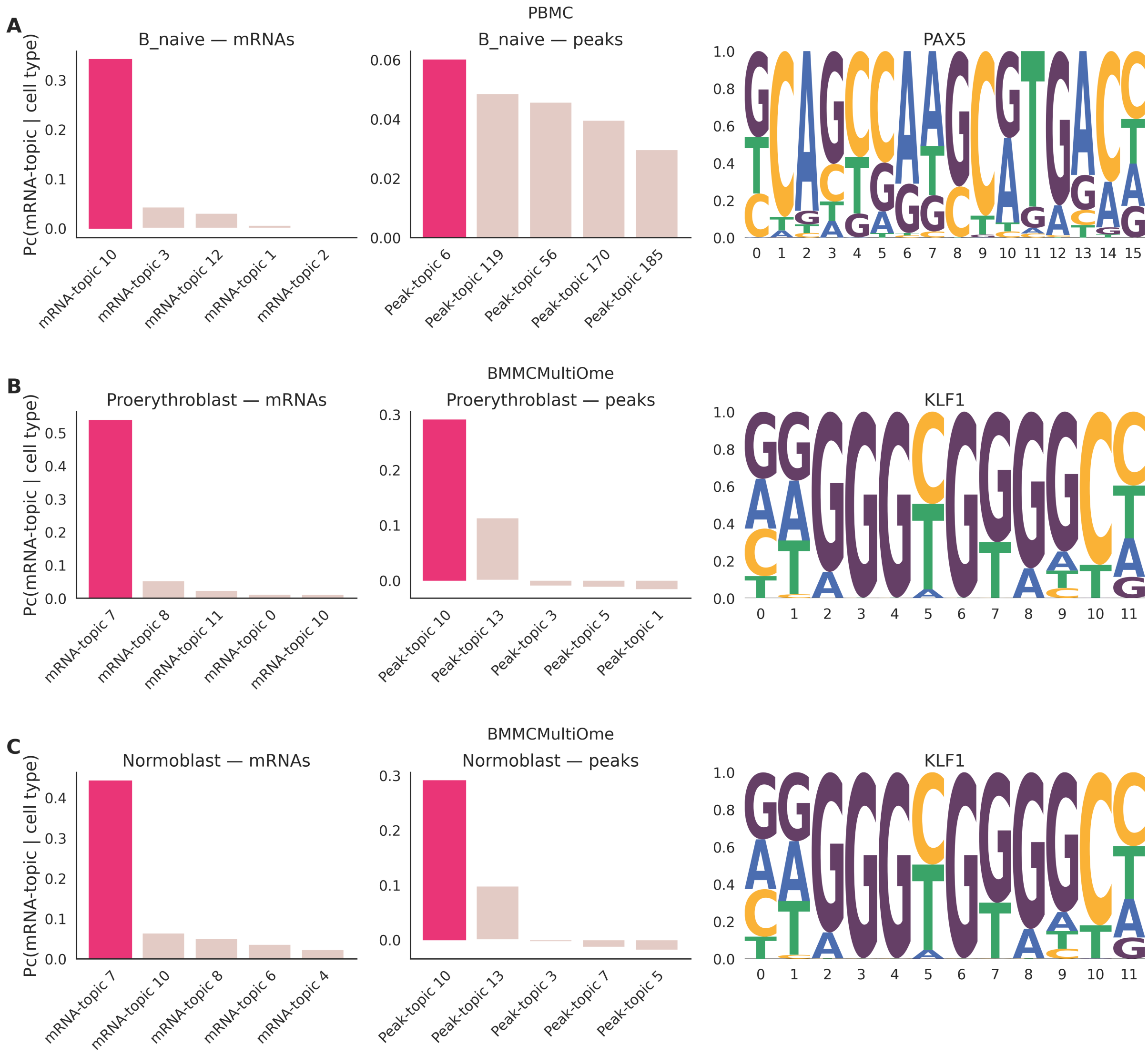

Fig. S6: For each data set, three panels are shown. **Left**: enrichment profile of the mRNA-topic with the highest distinctiveness for the given cell type. **Middle**: enrichment profile of the peak-topic with the highest distinctiveness for the given cell type. **Right**: sequence logo of a significantly enriched transcription factor motif identified in peaks belonging to the selected peak-topic (HOMER analysis).

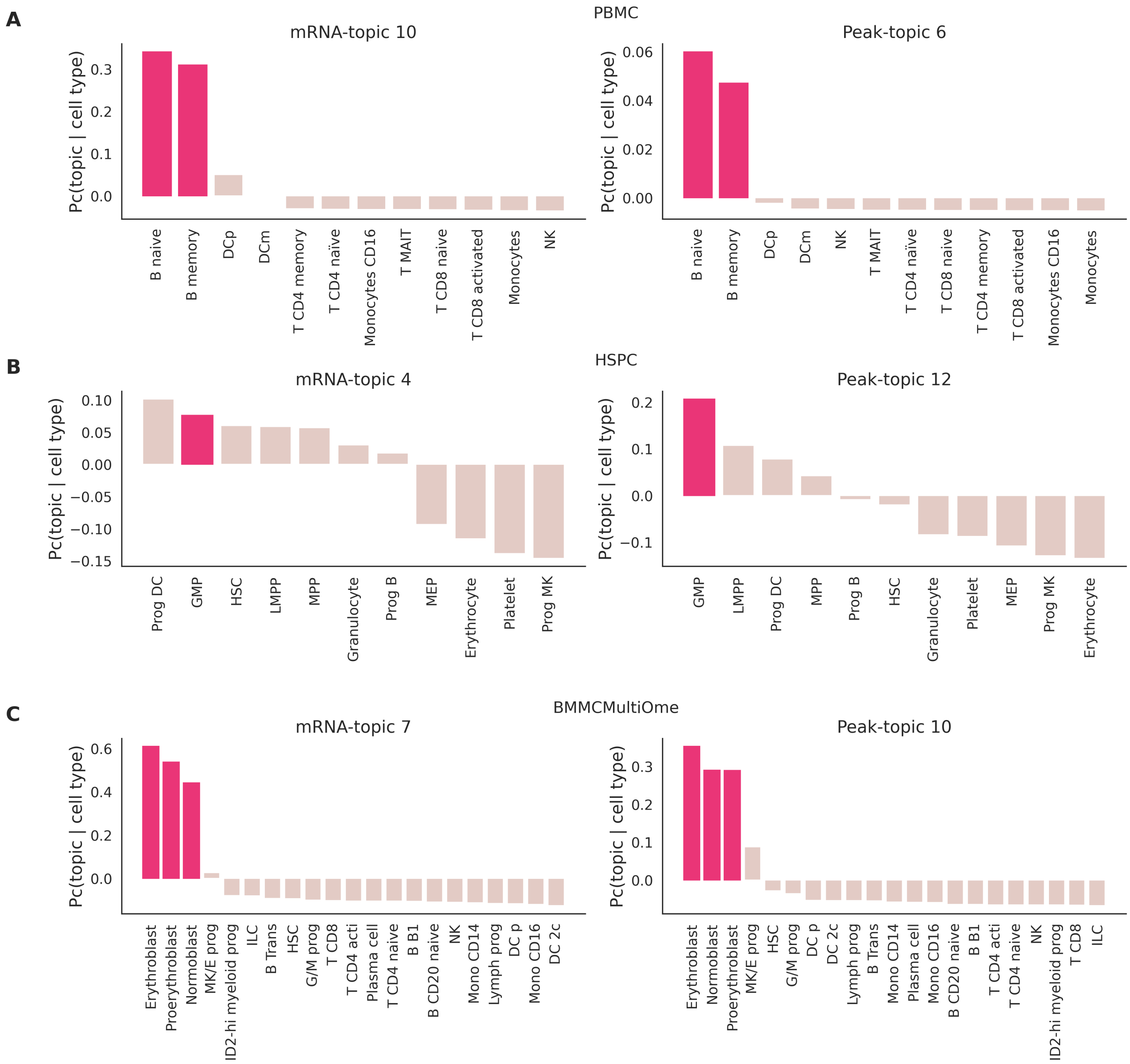

Fig. S7: **Left**: enrichment profile of the mRNA-topic with the highest distinctiveness for the selected cell type, shown across all other cell types. **Right**: enrichment profile of the Peak-topic with the highest distinctiveness for the selected cell type, shown across all other cell types.

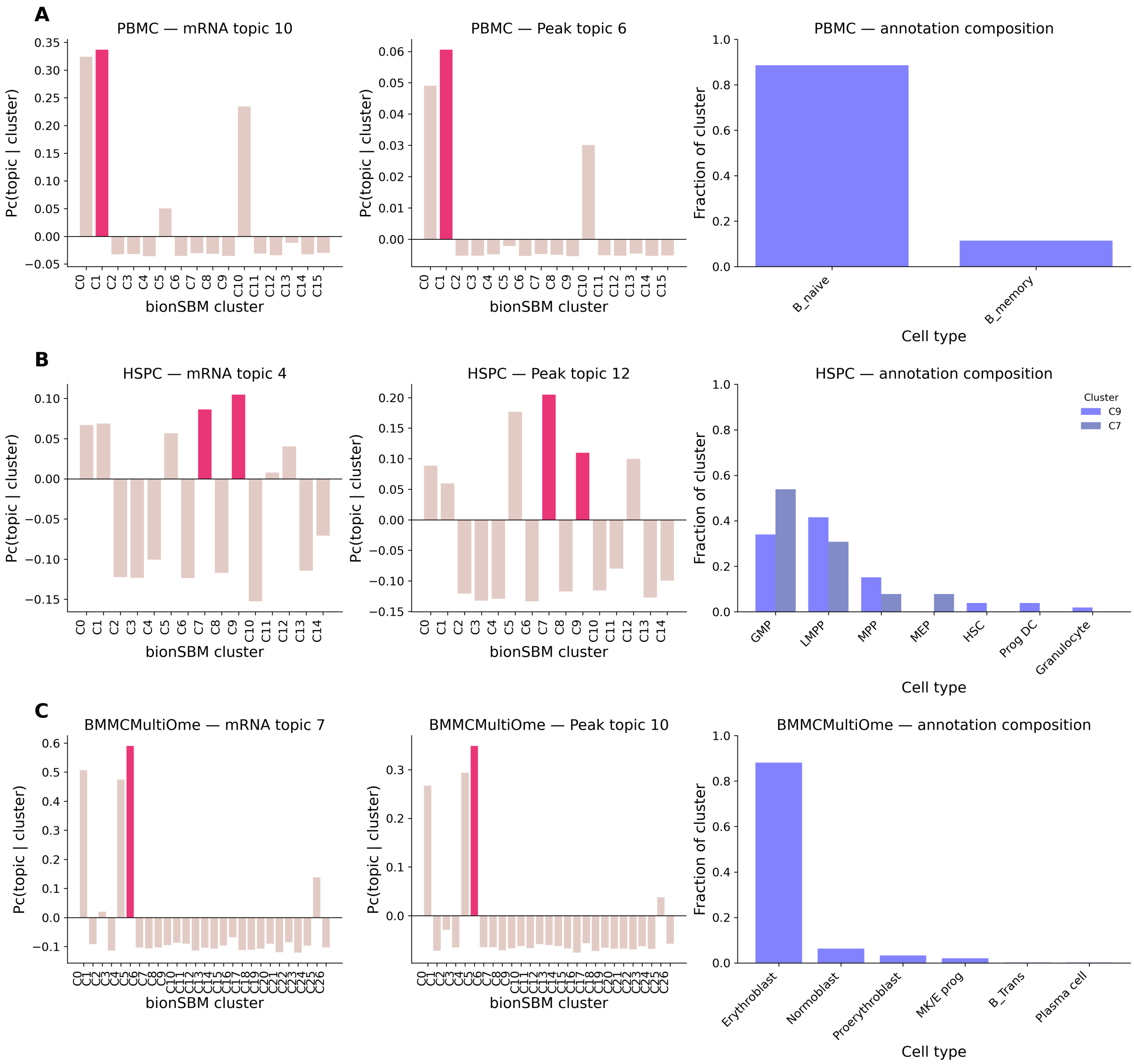

Fig. S8: **Left**: enrichment profile of the mRNA-topic with the highest distinctiveness for the selected cluster, shown across all other clusters. **Center**: enrichment profile of the Peak-topic with the highest distinctiveness for the selected clusters, shown across all other clusters. **Right** cell-type composition of the highlighted clusters.

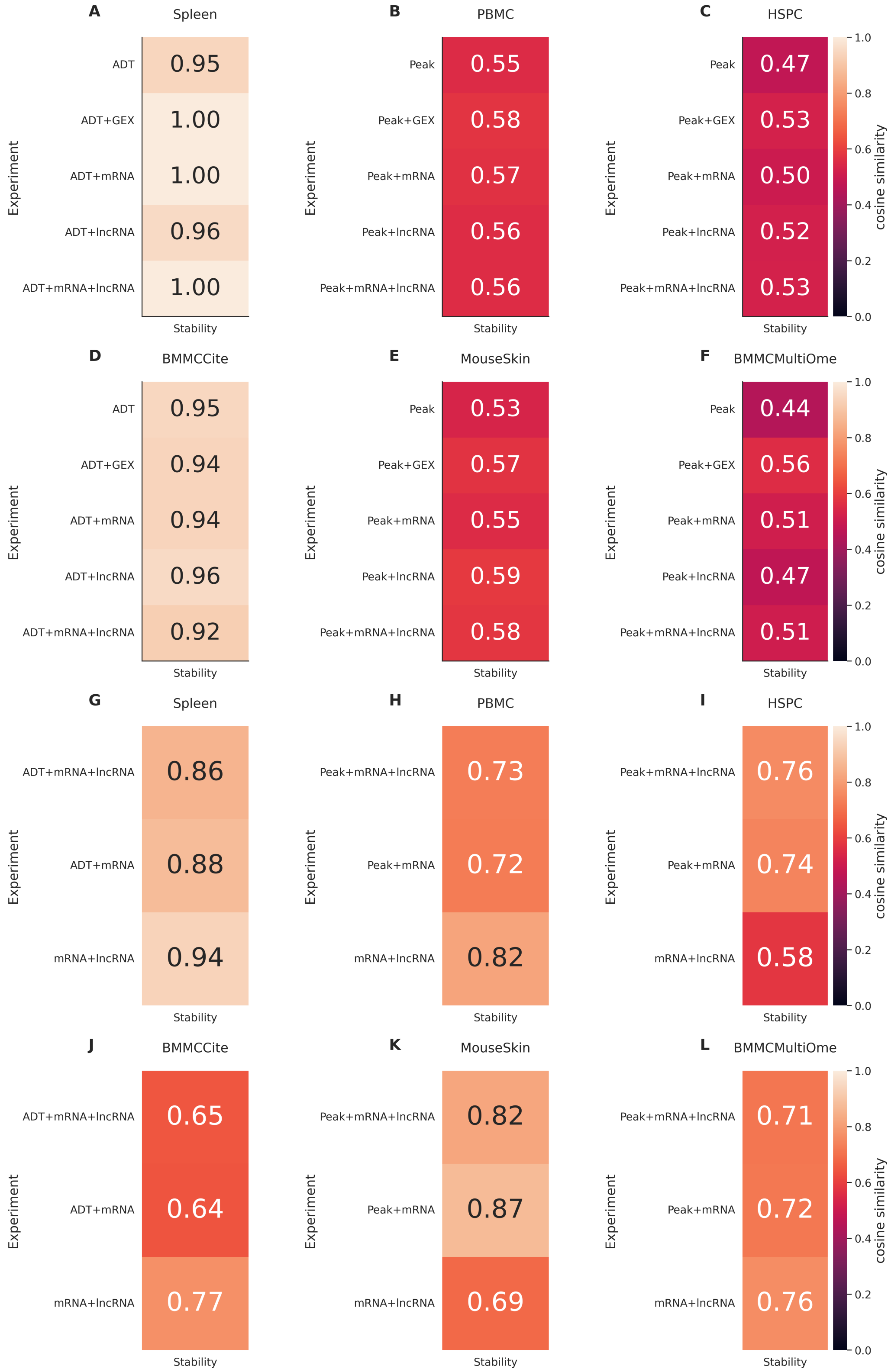

Fig. S9: Stability of the bionSBM algorithm. Average cosine similarity across 25 runs of the same experiment of the three most important ADT (**A**, **D**), peak (**B**, **C**, **E**, **F**) and mRNA (**G**–**L**) topics for each cell type.

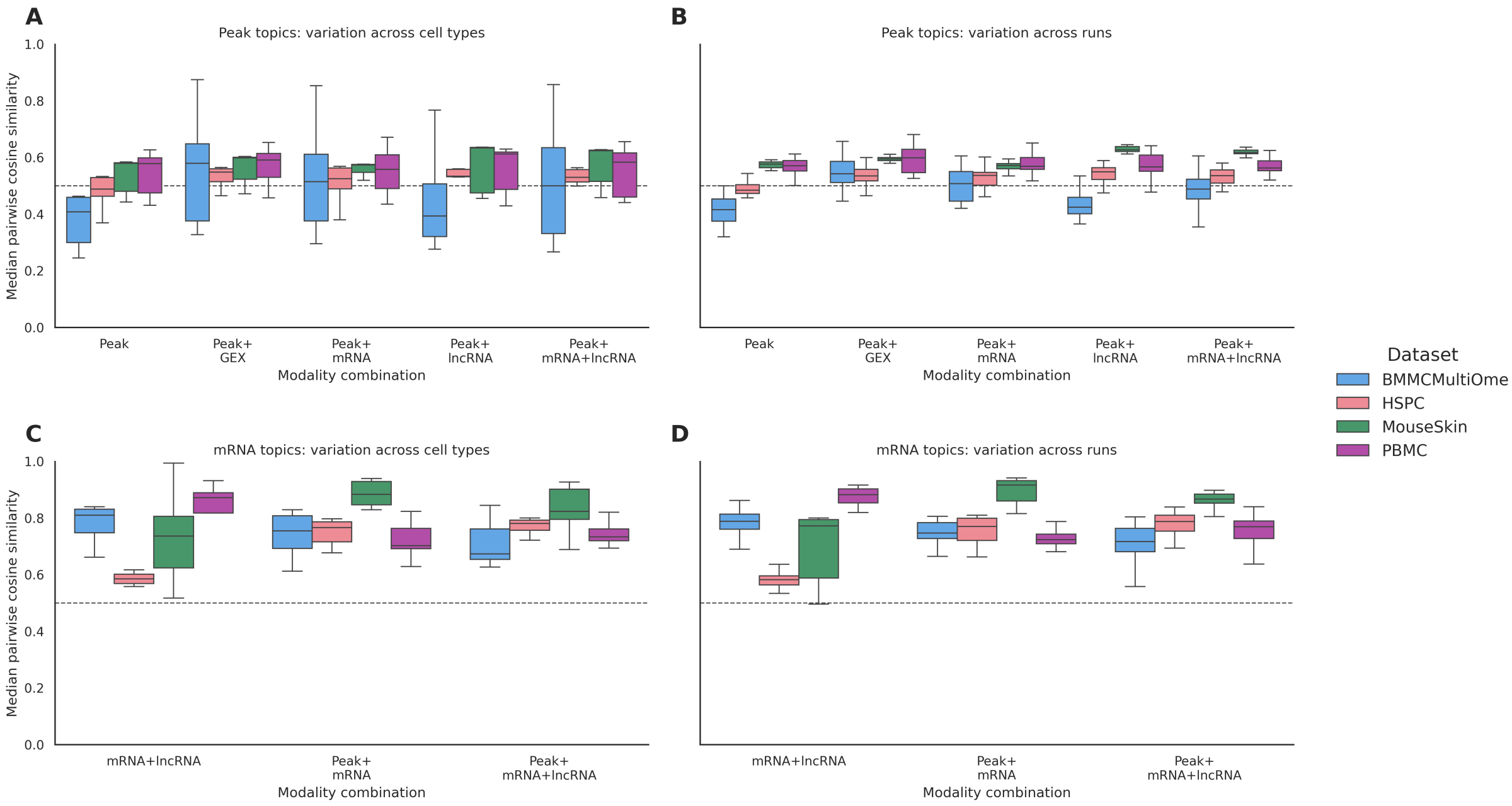

Fig. S10: Pairwise cosine similarity was calculated between the top three dominant topics identified in each pair of bionSBM runs. Distribution of cell-type-specific stability for Peak topics (**A**) and mRNA topics (**C**). For each cell type, cosine similarities were summarised across all pairs of runs, and the resulting median values are shown. Distribution of run-specific stability for Peak topics (**B**) and mRNA topics (**D**). For each run, cosine similarities were summarised across all comparisons involving that run and across cell types. Boxes are grouped by modality combination and colored by dataset.
